# Recovery support services as part of the continuum of care for alcohol or drug use disorders

**DOI:** 10.1111/add.16751

**Published:** 2025-01-28

**Authors:** Ed Day, Laura Charlotte Pechey, Suzie Roscoe, John F. Kelly

**Affiliations:** ^1^ Institute for Mental Health, School of Psychology University of Birmingham Birmingham UK; ^2^ Department of Health and Social Care Office for Health Improvement and Disparities London UK; ^3^ Harvard Medical School and Center for Addiction Medicine Recovery Research Institute, at Massachusetts General Hospital Boston MA USA

**Keywords:** alcohol, collegiate recovery program, drugs, employment support, peer‐based recovery support services, recovery check‐up, recovery coaching, recovery community centre, recovery high school, recovery housing

## Abstract

**Background:**

The definition of ‘recovery’ has evolved beyond merely control of problem substance use to include other aspects of health and wellbeing (known as ‘recovery capital’) which are important to prevent relapse to problematic alcohol or other drug (AOD) use. Developing a Recovery Oriented System of Care (ROSC) requires consideration of interventions or services (Recovery Support Services, RSS) designed to build recovery capital which are often delivered alongside established treatment structures. Lived experience and its application to the process of engaging people, changing behaviour and relapse prevention is an essential part of these services.

**Aim:**

To map out the evidence base for RSS as part of guidance for commissioners of addiction services in each of the 152 local authorities in England.

**Methods:**

The authors updated the findings of a 2017 systematic review of RSS through a further rapid scoping review, aiming to map out the extent, range and nature of research under six headings: (1) Peer‐based recovery support services (P‐BRSS); (2) Employment support approaches; (3) Recovery housing; (4) Continuing care and recovery check‐ups; (5) Recovery community centres (RCC); and (6) Recovery support services in educational settings. A systematic search of the PubMed, Embase, CINAHL, CENTRAL and PsychINFO databases was conducted. The abstracts of all articles published since 2017 were reviewed by two of the authors, and the full text versions of all relevant articles were obtained and relevant data extracted. A narrative review of the findings was then prepared, mapping them on to the ROSC continuum of care. The review was restricted to adults (over 18 years), but all substances and available outcomes were included.

**Results:**

Four of the six forms of RSS were well supported by evidence. RCTs of interventions to increase levels of employment demonstrated large effect sizes, and continuing care interventions that extend treatment intervention into the early recovery phase have shown small but significant benefit. Peer‐delivered interventions to link people to ongoing support were associated with decreased rates of relapse and re‐admission, increased engagement, and increased social support for change. However, the variability in the design of these studies means that further work is required to clarify the effective components of the intervention. Studies of recovery housing have also shown positive results, including significant differences from standard care. No controlled studies exist to support RCCs or RSS in educational settings, but the complexity of these interventions and the wide range of potential outcome measures mean that other study designs may be more relevant.

**Conclusions:**

This monograph provides a structure to help policy makers, commissioners and service providers describe and understand an emerging field of research. Recovery Support Services (RSS) are proving to have clinical, public health and cost utility. A rational social and fiscal response to endemic alcohol or other drug challenges should therefore include the more intensive acute care clinical services linked with more extensive community‐based RSS.

## INTRODUCTION

Regular use of psychoactive substances drives neuroadaptive changes in the structure and function of the human brain that underpin a transition from controlled, occasional substance use to more impulsive and compulsive, chronic use [1, 2]. Changes in brain circuits associated with reward, memory, motivation, impulse control and judgement may persist long after an individual stops using substances, and can produce continued, periodic craving for the substance. The drive to use substances is influenced by re‐exposure to places, people, times of day or mood states that have become strongly associated with alcohol or other drug (AOD) use through the process of classical conditioning. Furthermore, the historical long‐term prioritization of AOD use over other activities also means that an individual is often left with deficits in educational attainment, employment skills and social relationships, leaving them isolated from family and friends, unable to access safe housing and without viable job skills. The frequent stigmatization or criminalisation of AOD use compounds the problem.

Longitudinal studies have repeatedly demonstrated that treatment for AOD use disorder (particularly for 90 or more days) is associated with major reductions in substance use, physical and mental health problems and costs to society [3, 4, 5, 6, 7]. However, relapse after discharge from treatment and eventual re‐admission are also common [8, 9, 10, 11]. Even after sustained remission is achieved it can take another 4 to 5 years before the risk of meeting criteria for AOD use disorder in the next year drops below 15%, the annual risk for substance use disorder (SUD) in the general population [12, 13, 14, 15, 16]. These significant and lasting changes in brain neurobiology and the high rates of lapse and relapse mean that severe AOD use disorder should be treated as a chronic condition similar to asthma, diabetes and hypertension [17].

In general health care, treatments that reduce the symptoms of a chronic disease to ‘subclinical’ levels are said to produce remission. Serious AOD use disorders can involve cycles of abstinence and relapse over several years once remission has been achieved. An episode of treatment that leads to abstinence cannot be said to be a cure. Instead, continued remission may require ongoing monitoring and recovery management support, with early re‐intervention if the features of the disorder re‐occur [18, 19, 20]. Kelly and Hoeppner [21] have proposed a bi‐axial formulation of the recovery concept. The key substance‐related component (‘remission’) is placed on one axis and the positive consequences ensuing from, as well as supporting, the achievement of remission are placed on the other axis (‘recovery capital’). There is a reciprocal relationship between remission and recovery capital whereby initial remission leads to positive consequences and accrual of more recovery capital, and facilitating access to more recovery capital increases the chances of ongoing remission. The term recovery capital was introduced by Granfield and Cloud [22] and may be defined as the ‘resources and capacities that enable growth and human flourishing’ [23, 24]. Deficits in resources crucial to sustaining remission from AOD use disorder can create hopelessness, reduced motivation and a lack of resolve and ability to meet the demands of early recovery. A key organising principle of treatment and recovery services is, therefore, to build recovery capital, whether or not the individual has chosen to seek abstinence from substances.

Recovery‐oriented systems of care (ROSC) arose out of the shortcomings of an acute care model of addiction treatment. ROSC is an umbrella concept that represents the ‘entire network of formal and informal relationships and organizations that foster individual, familial, and community recovery processes over time’ [25]. The ROSC framework was designed to support the coordination of specialist and non‐specialist services and community networks and groups supporting recovery from AOD use disorders [26, 27]. Treatment and recovery services including peer‐led support providers [known as lived experience recovery organisations or LEROs in the United Kingdom (UK) [27]] are organised into a framework that incorporates the whole health and social care system. The ROSC should be easy to navigate, transparent and responsive to the cultural diversity of the community in which it operates. Two prominent examples of ROSC development have been described in the United States (US), one in a city (Philadelphia) [28] and the other at state level (Connecticut) [29]. When treatment is combined with long‐term recovery support, outcomes improve dramatically [30].

**FIGURE 1:**
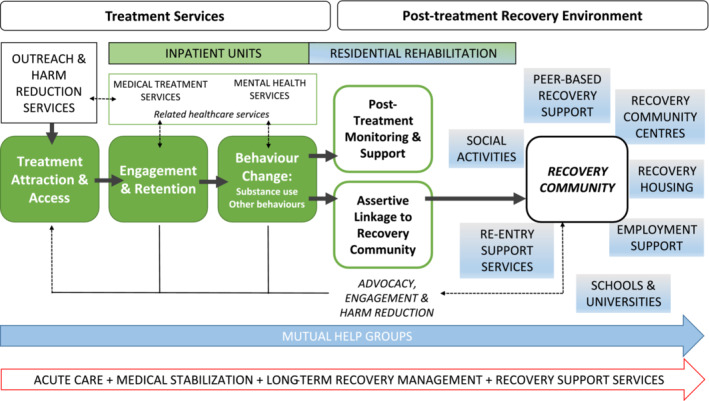
Diagram of the recovery oriented system of care (ROSC) used to inform the 2022 United Kingdom drug strategy ‘From Harm to Hope’. The left‐hand side of the diagram shows the stages of treatment system, and the right‐hand side shows recovery support services. Limited space precludes the inclusion of other key elements of the system, for example, primary care services, criminal justice services (e.g. prison, probation, police), social services (child protection and family support agencies), housing agencies and job centres. The diagram is schematic for two reasons: first, it represents a recovery journey as a straightforward linear series of events instead of a highly individual journey that may involve movement backward, forward and sideways. Second, it assumes that everyone with a significant addiction issue uses professional services or mutual help groups, when population survey data suggests that 50% of people achieve ‘natural recovery’ [33].

In 2020, the UK Government commissioned a review of specialist treatment services for drug use disorders [31], and subsequently produced a new national strategy document in response to the findings [32]. This emphasised the importance of a ROSC, and Figure 1 shows a schematic diagram developed by one of the authors (E.D.) to inform thinking about the commissioning of services in the 2021 strategy. To deliver a comprehensive ROSC, a full range of harm reduction, engagement, behaviour change and recovery support interventions must be described, commissioned, sequenced, implemented and evaluated [34]. Although professionally led treatment services were well developed in each local authority in England (Scotland, Wales and Northern Ireland have their own devolved commissioning process), the right‐hand side of the diagram emphasises the need for recovery support services (RSS) [26, 27, 35]. RSS may be defined as the array of interventions that support people to attain and sustain recovery in the community long‐term, building on the gains made in treatment if they have accessed it, and include support to deepen their connection to recovery communities and other resources. The variety of established and emerging RSS are intended to provide or facilitate increases in recovery capital, which in turn influences resilience and coping, and helps buffer and reduce stress.

RSS have been gradually defined in academic and policy literature over the past 25 years. The authors of this monograph set out to provide guidance for commissioners of services in each of the 152 local authorities in England. Its basis was a systematic review by Kelly [36], where the authors chose what they considered to be the six most important forms of RSS: (1) clinical models of continuing care; (2) peer based recovery support services; (3) mutual‐help organisations (MHOs); (4) recovery housing; (5) recovery community centres; and (6) RSS in educational settings. Early acute care models of treatment conceptualised Alcoholics Anonymous (AA) or other mutual‐help groups as the main form of RSS. However, MHOs were omitted from this monograph for two reasons. First, the evidence base for these approaches has been extensively reviewed elsewhere [37], showing enhanced and extended remission rates and high levels of cost‐effectiveness. Second, MHOs are peer‐led, often anonymous, interventions that cannot be commissioned directly. Instead, it was decided to include interventions to increase employment, a key element of recovery capital.

To update the findings from Kelly's 2017 review, a further rapid scoping review was undertaken to examine the extent, range and nature of research activity. Such a rapid review does not describe research findings in any depth but, in the words of Arksey and O'Malley [38], is a ‘useful way of mapping fields of study where it is difficult to visualize the range of material that might be available’. The search strategies are reproduced in full in the Supporting information. The abstracts of all articles published since 2017 were reviewed by two of the authors (E.D. and L.C.P.), and the full text versions of all relevant articles were obtained. The following information was extracted from each study: study design, description of intervention, sample size, follow‐up period, primary substance(s) and substance use and related outcomes. In reviewing the evidence for this monograph, priority was given to RCTs, quasi‐experimental studies and other research and evaluation designs that include a comparison condition. If no, or insufficient numbers of, studies were found in the systematic search at this level of scientific rigour we considered the next tier of available evidence (single‐group prospective studies or single‐group retrospective studies, followed by cross‐sectional/descriptive and qualitative studies). The review was restricted to adults (over 18 years), but all substances and available outcomes were included.

**FIGURE 2:**
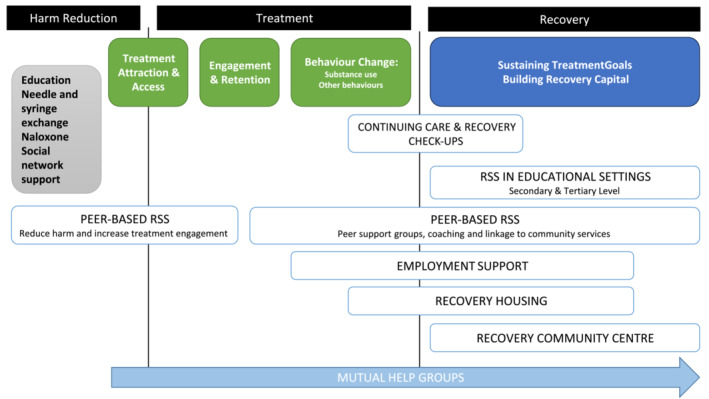
Potential sequencing of the six recovery support services across the continuum of care underpinning a recovery oriented system of care (ROSC).

The evidence presented is largely drawn from the United States and Canada, with surprisingly little published research conducted outside North America. We aimed to use the literature survey to highlight examples of evidence‐based practice, examine limitations of the evidence and describe ways in which policy makers, commissioners and service providers in the United Kingdom (specifically England) can use this evidence to develop effective ROSC. To assist the reader, Figure 2 places the six forms of RSS described in this monograph along the ROSC continuum of care shown in Figure 1 at the point in the recovery journey where they may have most impact: (1) peer‐based recovery support services (P‐BRSS); (2) employment support; (3) recovery housing; (4) continuing care and recovery check‐ups; (5) recovery community centres; and (6) RSS in educational settings. Some elements are very stage‐specific (e.g. P‐BRSS for treatment engagement) but others may be used at any stage (e.g. the resources of a recovery community centre). However, it should be noted that each individual recovery journey is different, and the neat linear form implied in Figures 1 and 2 would be unusual.

## PEER‐BASED RECOVERY SUPPORT SERVICES

‘Peer support’ may be described as ‘support provided by peers, for peers; or any organized support provided by and for people with (mental) health problems and illnesses’ [39]. Peer support roles are well established in the mental health field, where one‐to‐one peer relationships are associated with mutually positive benefits to both the recipient and the individual delivering the support [40, 41, 42]. The peer support approach assumes that people who have similar experiences can better relate to, and effectively establish trust and rapport with, those in need of support, leading to better engagement and retention in services. Peers act as role models for recovery allowing others to feel more hopeful about their own situation [43]. Support provided by people with lived experience of addiction recovery can be delivered through various organisational structures and service roles (including volunteer and paid roles). However, there are substantial differences between models of peer‐led recovery support and clinically led addiction treatment services.

Professional AOD treatment services are provided by individuals with formal training in a clinical setting. In contrast, P‐BRSS are mentoring, education and support interventions delivered by individuals who are ‘qualified by experience’ of their own journey through addiction and recovery. They differ from MHOs such as the 12‐step fellowships by not using a particular method of achieving and sustaining recovery, and not necessarily requiring abstinence. The core functions of P‐BRSS span all components of the ROSC including harm reduction, engagement, initiation and stabilization, recovery maintenance and long‐term recovery and may involve providing support at individual, family and community levels [44, 45]. Consistent with a chronic disease model of addiction, there is a focus on immediate recovery‐linked needs, use of self as a helping instrument and continuity of recovery support over time. This might be in addition to, or instead of, professionally driven treatment [46].

### Challenges of peer support roles

The benefits for individuals of delivering a peer support role include increased confidence, stability, structure, income and an opportunity to gain workplace skills [47]. People with lived experience of addiction working in peer specialist roles can offer strong personal connection, encouragement and hope [48]. Personal experience of the challenges of addiction and recovery may make them easier to relate to than clinical roles, and shared experience may help to overcome the inherent power differential between clinicians and people accessing support. For example, peer workers can occupy a unique space in the acute care hospital setting through their ability to form meaningful relationships with hospitalized patients quickly, based on trust and shared lived experiences [49].

However, there are many challenges to implementation in healthcare settings, including the hierarchical staffing structure of hospitals and treatment services, stigma and discrimination toward people with SUD, inconsistent and unclear role descriptions and fast‐paced work with high demand [50]. P‐BRSS often sit at the interface between purely peer support and purely clinical support roles. Peer support workers may not have the clear differentiation between ‘staff member’ and ‘patient’ enjoyed by hospital and treatment staff, whilst also being discouraged from following the self‐regulating, volunteer‐led traditions of mutual help roles [36]. Although peer workers often feel their role leads to professional and personal development, the intensity of working in some settings (e.g. hospital) also exposes them to emotional stress [49]. A lack of organizational understanding and recognition of these roles is evident from unclear ‘peer’ role titles, a lack of role communication and expectations, the representation of experiential knowledge and a lack of role support and training [51]. Low pay has also been cited as a source of major dissatisfaction with these new roles [52]. P‐BRSS roles may be improved by attention to the needs of supervisors in terms of skills for effective supervision and clarification of supervisory roles [53].

### P‐BRSS to reduce harm and increase engagement in treatment

There is a long tradition of peers engaging other people who use drugs (PWUDs) and linking them to treatment and recovery support, while also reducing the harms associated with active substance use [54]. Early attempts at delivering harm reduction services were often unsanctioned peer‐led initiatives, and this approach extended the reach of needle and syringe exchange programs to new populations [55, 56], or improved levels of user satisfaction and mental health [57]. A review of evidence for the role of PWUD in harm reduction initiatives has described an array of cross‐sectional and short‐term prospective evaluation studies focused on education; distributing needles/syringes, condoms and injecting paraphernalia; blood borne virus screening, counselling and treatment; or operating 24‐hour information lines [58].

There has been a recent focus on the distribution of naloxone to reverse accidental opioid overdose [59], and members of the public (as opposed to just health care staff) may lawfully possess and administer naloxone for the purpose of saving life in many countries. PWUD are motivated and willing to administer naloxone if they witness an overdose, and a systematic review and descriptive meta‐analysis of take‐home naloxone (THN) programmes for people who use opioids found that nearly 10 kits are used for peer administration for every 100 people trained [60]. Naloxone provision is often combined with education as part of an opioid overdose prevention education and naloxone distribution (OEND) program. Training includes brief interventions, peer education and overdose management and intervention (e.g. administering naloxone). A systematic review of the evidence for peer support and overdose prevention responses [61] highlights that OEND programs improve knowledge and increase the likelihood of active overdose response behaviour among PWUD [62, 63]. Peer‐delivered overdose prevention is a viable way to reach PWUDs who are reluctant to seek medical help [64], and providing PWUD with naloxone is recognized as one element of a comprehensive overdose prevention strategy [65, 66].

The opioid crisis in North America has led to innovative peer‐based strategies to overcome barriers to accessing harm reduction services or opioid agonist treatment (OAT) [67]. Such approaches have received widespread federal and state funding in response to a high death rate from opioid overdose, and since 2017 programs have been evaluated in Florida [54, 68], Pittsburgh [69], New Jersey [70], Vancouver [71], Rhode Island [72, 73], Indiana [74] and Nevada [75, 76]. For example, a pilot RCT (*n* = 80) evaluated a single 20‐minute peer‐delivered telephone intervention for preventing recurring opioid overdoses [77]. All participants received personally tailored OEND following an opioid‐positive urine drug screen, but the peer support group got a 20‐minute intervention from a trained peer with the goal of engaging the participant in OAT. At the 12‐month follow‐up 32.5% of the peer support group enrolled in OAT compared to 17.5% of the control group (OR = 2.27, 95% CI = 0.79–6.49). Peer support group participants were significantly less likely to have experienced an overdose than those in the control group (12.5% vs. 32.5%, *P* = 0.03). However, no significant objective or self‐reported treatment effect was found for opioid use at 12‐month follow‐up [77].

### Peer support groups, coaching and linkage to community services

P‐BRSS may (1) address multiple life domains impacted by AOD use; (2) help individuals transition through the continuum of care; and (3) help individuals sustain recovery after formal treatment has ended through skills training and coaching [78]. A review of evidence from both the mental health and addiction fields found that peer support services produced positive outcomes in two broad areas [41]: (1) personal skill building (i.e. peer support can foster hope, build motivation, self‐awareness and recovery values and help to prevent relapse and enable self‐management of addiction); (2) community skill building (i.e. by improving interpersonal relationships peer support can facilitate engagement in services and community affiliation and support employment). P‐BRSS have an important role in linking people into ongoing support and building recovery capital when formal treatment has ended.

Peer support roles have been described and evaluated in a variety of different settings and populations, including detoxification units [79], acute hospitals [49, 50, 80], perinatal services [81], primary care [82, 83], homeless populations [84], the criminal justice system [80, 85, 86, 87, 88], young people [89, 90, 91] and minority ethnic groups [92]. Systematic reviews of P‐BRSS including RCTs, quasi‐experimental and single site cohort studies have shown an association between P‐BRSS interventions and reduced substance use and AOD dependence relapse rates, increased treatment retention and greater treatment satisfaction [36, 93, 94]. In the largest study (*n* = 1175), conducted in people using cocaine and/or heroin presenting to a hospital‐based walk‐in clinic, participants receiving a single session of peer support were more likely to be abstinent than the control group for cocaine alone (22.3% vs. 16.9%), heroin alone (40.2% vs. 30.6%) and both drugs (17.4% vs. 12.8%) 6 months later (adjusted OR = 1.51–1.57) [95]. Three smaller RCTs [96, 97, 98] (*n* = 114, 96 and 137, respectively) involving people with co‐occurring severe mental illness and substance use tested P‐BRSS interventions of differing design, intensity and duration, with similar results. Despite the promise of peer‐delivered interventions, this literature has significant methodological limitations including an inability to distinguish the effects of peer support from other recovery support activities, heterogeneous populations, and inconsistent definitions of peer workers and lack of appropriate comparison groups [93].

One method of delivering peer support is recovery coaching, which is described as a continual model of care tailored to an individual's specific needs, recovery stage and personal challenges [99]. Recovery coaches are peer workers who address barriers to building recovery capital such as housing, financial problems, legal issues or accessing healthcare services. The approach is tailored to individual need and can work alongside medication, mutual aid meetings and psychological interventions [94]. For example, a prospective RCT compared outcomes between the standard of care and a recovery coaching intervention in 98 participants who had been hospitalized because of AOD complications [99]. Recovery coaches delivered 10‐ to 120‐minute sessions 1 to 2 times per week using motivational interviewing, providing coping strategies and offering emotional, social and familial support. Engagement over the 6 months post‐discharge was higher for participants in the recovery coaching intervention (84%, 95% CI = 78%–91%) compared to the control condition (34%, 95% CI = 25%–44%, *P* < 0.001). Systematic reviews have provided further preliminary evidence to support the effectiveness of recovery coaching‐style interventions [93, 94, 100], demonstrating an association with reduced rates of relapse and re‐admission, increased social support and increased retention in mutual‐help groups. However, these conclusions are tempered by methodological limitations such as the lack of clarity in descriptions of the intervention(s) delivered and the training protocols that coaches received, and a lack of appropriate comparison groups and definitive outcomes [100].

### Policy and practice implications

Peer worker roles and services have developed to target particular points in the care continuum [44, 45], and an evidence base is emerging for specific interventions delivered by individuals that use their lived experience of addiction and recovery. Describing the components of P‐BRSS (e.g. OEND and recovery coaching) helps policy makers, commissioners and providers of services understand how to deploy these interventions in the most effective way across the ROSC. The research literature provides plenty of qualitative support for the benefits of P‐BRSS, but further controlled studies are required to tease out the distinct effects of P‐BRSS from other treatment activities [36, 93, 94].

In England, a 2023 national treatment and recovery workforce census indicated that peer support roles made up 8% of the treatment provider workforce, with 75% of these roles unpaid [101]. The 10‐year drug and alcohol treatment and recovery workforce strategic plan has delineated a ‘peer support worker’ role, acknowledging that such a job description is different to clinical and volunteer roles and requires its own standards around training, supervision and support [102]. The specific capabilities of this role are being defined, and a new training curriculum for peer support workers is also being developed. The United Kingdom has also identified the added benefits of peer‐led organisations that grow out of local communities (known as LEROs) [27], and the 2021 drug strategy ‘From Harm to Hope’ committed to encouraging the development of a network of recovery organisations [32].

The challenges faced by people in peer support worker roles include hierarchical staffing structures, stigma and discrimination, poorly developed job descriptions and risk of relapse to uncontrolled AOD use [50]. As recognised in the workforce strategic plan for England, peer support workers should ideally be managed and supervised by a suitably trained and experienced person, ideally with lived experience themselves, to maximise the chances of overcoming these barriers. UK context‐specific research would help clarify P‐BRSS role definitions, identify optimal training guidelines for peer support workers and establish for whom and under what conditions P‐BRSS are most effective.

## EMPLOYMENT SUPPORT

Research shows a strong negative relationship between AOD use and employment [103], whereas stable work generates income, fills and structures time, provides access to new social groups and is associated with better mental and physical health [104]. Being employed is one of the strongest predictors of positive outcomes for people with AOD use disorder, including more frequent treatment completion, lower incidence of relapse, less criminality and improved quality of life (QoL) [105, 106]. Many people receiving treatment for AOD use disorder can and want to work, but struggle to access the open job market and achieve stable employment. Approximately three‐quarters of people in treatment in the United States and United Kingdom report being unemployed, and the numbers who find work during or after treatment are low [107, 108]. Engagement in standard outpatient AOD treatment services has not been shown to increase rates of employment [109], but unemployment increases the risk of relapse after AOD treatment [103]. Treatment without additional support to find work, housing and develop life skills has limited and inconsistent effects on employment [110, 111]. Barriers to work for people with AOD use disorder include a lack of education and skills, poor physical and mental health, social disadvantage and stigma [112, 113].

Magura and colleagues [109, 114] have published two systematic reviews of the evidence for interventions intended to improve employment outcomes for people over 18 years with AOD use disorder, covering the periods 1981 to 2004 [109] and 2005 to 2018 [114]. The latest review included 13 articles representing eight different programs for adults that used a minimum of a quasi‐experimental evaluation design. Of the interventions with specific employment support as part of the intervention, Individual Placement and Support (IPS) and its variant Customized Employment Supports (CES) had the most studies with positive results [115, 116, 117, 118, 119]. Other approaches were Therapeutic Workplace (TW) [120, 121], Drug Court Employment Intervention [122], Job Seekers Workshop (JSW) [123] and integrated interpersonal cognitive problem solving [124].

*Individual Placement and Support* (IPS) is delivered by trained employment specialists (ES) as part of a multi‐disciplinary clinical treatment team, rather than separately provided by a generic job seeking service. IPS aims to get anyone who wants to work into their preferred type of employment in the open market, with no exclusions based on diagnosis or perceived complexity. The ES provides time‐unlimited, individualised support for both the individual and their employer. Research evidence for the benefits of IPS has accumulated in different populations [125]. A series of studies in the US Veterans Administration services have shown a positive impact in homeless populations [126], and in veterans with spinal cord and traumatic brain injury [127] and post‐traumatic stress disorder [128]. A meta‐analysis of the findings from four RCTs comparing IPS to other established vocational approaches for people with severe mental illness strongly favoured IPS, with large effect sizes for job acquisition, total weeks worked and job tenure [129]. These results were achieved regardless of background demographic, clinical and employment characteristics. IPS has subsequently been shown to have a beneficial impact in people with first episode psychosis [130].

Significant differences in work‐related outcomes have been shown in US IPS studies where most of the sample had a diagnosis of current or past AOD dependence. For example, 45 patients receiving OAT for more than 6 months were randomly assigned to IPS or a waiting list control group. At 6‐month follow‐up, 50% of individuals in IPS were employed versus 4% of those on the waiting list (*P* < 0.001) [116]. In a comparison between an IPS cohort (*n* = 321), and a historical comparison cohort receiving usual care (*n* = 308) followed up for 2 years, the IPS cohort achieved a mean of 8.4 days/month of competitive employment versus 7.3 days/month for the comparison cohort (*P* < 0.001) [118]. A secondary data analysis combining subjects with co‐occurring mental illness and AOD disorders from four separate RCTs of IPS (*n* = 47) versus a comparison program condition (*n* = 59) found that 60% of IPS group obtained competitive jobs at 18‐month follow‐up compared with 24% of the comparison group (*P* < 0.001). Furthermore, individuals in IPS worked a total of 366 hours compared with 84 hours in the comparison group (*P* < 0.001) and had average total wages of $3050 [compared with $807 in the comparison group (*P* < 0.001)] [117].

*Customized Employment Supports* (CES): This is adapted from IPS, but tailored for people receiving SUD treatment, emphasizing small caseloads, therapeutic alliance, rapid job search, vocational fieldwork and long‐term support [131]. An RCT implemented at two methadone treatment programs in New York City found 10% of drug‐free individuals randomized to CES were employed at 6‐ to 12‐month follow‐up, compared with 6% of those in standard treatment (n.s. = not significant). In total, 41% of individuals in CES obtained any paid employment versus 26% of those in standard treatment (*P* < 0.05) [114].

*Therapeutic Workplace* (TW): Many people continue to participate in mainstream social and economic activities despite an AOD use disorder, and this is a potentially stabilising force in their lives [108]. For example, empirical data from a population of people who inject drugs resident in inner‐city Vancouver suggested that 30% participated in the labour market at any time [132]. Employment has been used by employers as an incentive to promote drug abstinence, a form of contingency management offering individuals a tangible benefit for providing objective evidence of drug abstinence [133, 134]. In the TW adults with a history of AOD use disorder are hired to work but required to provide drug‐free urine samples to gain access to the workplace and/or to maintain their maximum rate of pay. RCT evidence supports the TW as effective, relative to control conditions, in promoting abstinence from alcohol in homeless, alcohol‐dependent adults [135]; cocaine abstinence in participants receiving methadone treatment [136]; abstinence from opiates and cocaine in people injecting [137]; and adherence to naltrexone in adults with opioid use disorder [138, 139, 140]. More recently, the TW wage supplement program has been shown to be effective at promoting opiate and cocaine abstinence, increasing employment and reducing poverty [141], in addition to helping participants develop academic and employment skills and improve mood and QoL [142, 143]. Despite the cost involved in rewarding contingencies there is some evidence that the costs are comparable to other addiction treatments [144]. However, all the above RCTs were conducted by the same academic group in small sample studies (*n* = 33–124), and the research team notes that relapse to drug use and unemployment is usual when incentives are discontinued [145].

*Integrated Interpersonal Cognitive Problem Solving* (IICPS): A National Institute on Drug Abuse‐funded project that developed a manual‐based intervention that integrated drug counselling and employment services, trained methadone treatment staff in the intervention and conducted a pilot RCT (*n* = 23) to evaluate the intervention in a real‐world community clinic [124]. IICPS was the theoretical underpinning, helping the participant think through their problems and select options to reach a realistic goal. The study found no significant difference at 6‐month follow‐up in rate of employment or average monthly income between employment intervention and counselling‐only control, but a quarter of participants were lost to follow‐up at 6 months.

*Drug Court Employment Intervention*: In order to target people involved in drug‐related crime, entrants to drug court on nonviolent charges were randomized to an employment intervention (*n* = 244) or drug court as usual (*n* = 233) [122]. At 12‐month follow‐up 61% of the participants had completed at least half of the sessions, and there were significant differences in past year days of paid employment (210.1 for intervention, 199.9 for control, *P* < 0.05). However, intervention effects were modest given the intensity of the intervention, possibly because of a ceiling effect on outcomes as half of the subjects were employed at baseline [122].

*Job Seekers' Workshop* (JSW): Developed in the late 1970s, this intervention was administered in three 4‐hour small group sessions focusing on locating available jobs, making ‘cold calls’ to potential employers and rehearsing job interview skills. Videotaped feedback was used to enable participants to practice and improve their job interview skills. An early pilot study (*n* = 49 people accessing a methadone clinic) found 50% of the JSW group obtained employment/training at follow‐up, compared to 14% of controls (*P* < 0.05) [146]. A second study in an opioid dependent population in the criminal justice system (*n* = 55) found rates of employment were significantly higher for JSW versus control subjects (86% and 54%, respectively, *P* = 0.01) at 3‐month follow‐up [147]. A later study compared participants randomized to JSW or standard care in a larger, heterogeneous sample including both methadone maintenance and psychosocial outpatient treatment. There were no group differences in rates of employment or training [123], and the research team suggested that either more intensive interventions were required or a focus on alternative targets such as building motivation or training in specific skills.

### Policy and practice implications

Interventions to increase and sustain rates of employment in people with AOD use disorders have been widely tested. Eleven of 13 studies aimed at adults reviewed by Magura *et al*. [114] reported statistically significant intervention effects for at least one employment‐related outcome. However, the magnitude of effects on employment was small in most studies, and it was rare that all the outcomes measured showed significance [114]. England has made some progress in implementing and evaluating one of these interventions (IPS) following a review of employment outcomes in patients of SUD treatment services [106]. The Individual Placement and Support‐Alcohol and Drug dependence (IPS‐AD) study was a pragmatic, multicentre RCT of IPS in England for people enrolled in community treatment for AOD dependence [148]. A total of 1687 participants were randomly allocated to IPS or treatment as usual (TAU). IPS was associated with an increase in attainment of employment compared with TAU (adjusted OR = 1.29), and returned an incremental quality‐adjusted life year outcome gain of 0.01 (range, 0.003–0.02) per participant with no evidence of cost‐effectiveness at two willingness‐to‐pay thresholds (£30 000 and £70 000) [148]. A process evaluation of the IPS‐AD trial found study participants had different levels of confidence and motivation around finding employment, varying levels of work experience and different personal circumstances, but they described improved confidence and motivation to work through the intervention [149]. Both participants and employment support workers found engaging with employers challenging because of perceived stigma about hiring people with SUD. Participants often disengaged from the IPS service after enrolment and assertive re‐engagement techniques using letters, text messages and phone calls were required. Services operating in rural areas found it particularly challenging to engage people and to find suitable employment. However, IPS was implemented successfully in all seven treatment services included in the trial, and it is currently being rolled out across England supported by government funding.

## RECOVERY HOUSING

The social environment is a key predictor of success at every stage of the recovery journey. Finding safe and stable housing can provide the impetus to start and maintain a period of treatment, and the Housing First initiative is an evidence‐based practice to address this issue [150, 151]. Supportive housing can also be a critical part of a package of ongoing abstinent recovery support. ‘Recovery housing’ is a form of RSS that is usually accessed following formal treatment, where individuals with a common experience of addiction live together in a safe and supportive environment that is free of AOD. It is estimated that 1.2% of individuals with SUD reside in recovery homes each year in the United States [152].

Many different models of practice exist, however, as a minimum, recovery housing offers peer‐to‐peer recovery support aimed at promoting abstinent long‐term recovery. The National Alliance of Recovery Residencies (NARR, https://narronline.org) is a non‐profit US organization that coordinates affiliate organizations supporting more than 160 000 persons in recovery across 6500 certified residencies. NARR has described four levels of care provided by different types of residences [153], which are summarised in Table 1. In addition to 24‐hour support, the level of on‐site services and input from trained staff into daily operations occurs across a spectrum: (1) houses that are entirely peer‐run (level 1, e.g. Oxford houses); (2) those with a house manager (level 2, e.g. sober living homes); (3) those that require simultaneous attendance at specialist outpatient treatment as a requirement of residence (level 3, e.g. recovery homes in Philadelphia); and (4) residential treatment providers (level 4) [154]. The following section describes the first three levels of housing, as the evidence for professionally led residential rehabilitation facilities (level 4) can be found elsewhere [155, 156].

**TABLE 1:** Characteristics of the four levels of recovery housing provision as defined by the NARR in the United States [153].

	Levels of support			
	Level 1: Peer‐run	Level 2: Monitored	Level 3: Supervised	Level 4: Treatment provider
Physical structure	Single family house	Single family residence Flat or multi‐family units	Varied	Varied Unit within a facility (e.g. prison)
Staffing	No paid staff	Paid resident house manager	Paid house manager Director and administrative support Certified peer support	Paid house manager Director and administrative support Certified peer support Qualified staff
Governance and oversight	Written policies and procedures Democratically run	Written policies and procedures Resident participation	Written policies and procedures Resident participation varies Organizational hierarchy Administrative oversight Regulated Peer support staff supervision	Written policies and procedures Resident participation varies Organizational hierarchy Administrative oversight Regulated (e.g. CQC) Clinical supervision
Services	Drug testing/breathalyser House meetings Household tasks or chores Self‐help group attendance encouraged Use of services encouraged	Drug testing/breathalyser House meetings Household tasks or chores Self‐help group attendance encouraged Use of services encouraged	Drug testing/breathalyser House meetings Household chores Self‐help group linkage Clinical service linkage Recovery support services Life skills services	Drug testing/breathalyser House meetings Household tasks or chores Self‐help group linkage/on‐site Clinical service linkage Recovery support services Life skills services
Example	Oxford House	Sober Living Home (California)	Philadelphia OAS‐funded recovery home	Therapeutic communities

Abbreviations: CQC, Care Quality Commission; NARR, National Alliance of Recovery Residencies.

### Level 1

Oxford houses (OH) are democratically run self‐supporting homes that have no time limit for how long a resident can live there while abstinent from AOD. They represent the largest single network of recovery houses in the United States with over 3000 homes in 493 towns and cities [157], and outcome research has been ongoing for over 20 years. Jason *et al*. [158] recruited participants before discharge from residential treatment and randomly assigned them to either an OH or ‘treatment as usual’ that was arranged by the participant (e.g. outpatient treatment, self‐help groups and alternative living arrangements). At the 24‐month follow‐up point those in the OH group reported significantly lower substance use (31.3% vs. 64.8%), significantly higher monthly income ($989.40 vs. $440.00) and significantly lower incarceration rates (3% vs. 9%) compared with the usual‐care condition [158]. When the costs of healthcare, criminal activity, imprisonment, substance use and employment were considered, the overall net benefit over 2 years was approximately $29 000 higher per participant for OH residents [159]. Younger residents that stayed at least 6 months had the best outcomes [160]. Data from the trial have also been used to explore the mechanisms through which OH may produce beneficial effects. For example, longer stays are associated with having more people in a social network who are in recovery [161], and beneficial effects may be further enhanced when recovery housing is combined with high levels of 12‐step mutual help participation [162]. Recovery homes may increase social capital by sharing bonds through friendships with abstinent peers [163] and by supporting abstinence self‐efficacy despite substance using family members [164].

A second RCT randomly assigned 270 individuals released from the criminal justice system to an OH, a therapeutic community (TC) or usual care settings (UA). Participants in the OH condition achieved significantly higher continuous rates of abstinence from alcohol (66%) than the TC (40%, *P* < 0.01) or UA condition (49%, *P* = 0.02), although there were no significant differences for rates of continuous drug abstinence (OH, 47%; TC, 44%; UA, 42%; *P* = 0.84) [165]. OH residents earned more money from employment, worked more days and had more favourable cost–benefit ratios than the other conditions [165].

### Level 2

Sober living houses (SLH) are AOD‐free residences for individuals wishing to maintain abstinence that emerged in California in the 1970s. They have no limit on length of stay, are financially sustained through resident fees and do not offer formal treatment, but strongly encourage 12‐step group attendance [166]. Like OH they exist outside the formal (regulated) treatment provider system, but many are members of SLH coalitions that monitor quality, health and safety. In contrast to OH, they usually have a ‘house manager’, often someone in recovery who rents out rooms, collects money for rent and bills and evicts individuals for relapse. However, SLHs adhere to a social model of recovery that prioritises peer support and 12‐step group involvement [167], and so most require evidence of resident involvement in day‐to‐day operations.

A longitudinal study of nearly 300 men and women entering 20 SLHs over 18 months found that homelessness declined from 16% to 4% and stable housing increased from 13% to 27%. Psychiatric severity was generally mild to moderate, but also showed improvement over the study period [168]. Social support variables predicted substance use outcomes for persons with low and moderate psychiatric severity [169]. SLH residency has also shown benefits for a population drawn from the criminal justice system. At 6‐ and 12‐month follow‐up, 330 people on probation or parole showed significant improvement relative to baseline on substance use, criminal justice, HIV risk and employment outcomes [170].

Factors associated with social model recovery (i.e. involvement in 12‐step groups and social network characteristics) have been found to be associated with outcomes of level 2 housing [171]. Operational characteristics such as where the house was located and whether the house required incoming residents to be sober for at least 30 days before entry were also related to improved outcomes [172]. Even after relatively short periods of time in SLHs, resident perceptions of the social environment show associations with increased objectively measured recovery capital [173].

### Level 3

Level 3 houses differ from levels 1 and 2 in that they are more likely to incorporate treatment components with paid staff and have time‐limited residencies. Peer‐reviewed evaluations are rare, but one example described 10 recovery houses in Texas that explicitly bridged treatment and peer support by providing a variety of RSS [154]. All residents met with a recovery coach, underwent regular drug screening and had access to intensive outpatient treatment from a service developed specifically to support their needs. Unlike the OH and SLH models, these homes were financed by a mix of resident fees and private and public insurance, and residents had a limited role in the governance of the homes. A second study used administrative and qualitative data from an outpatient substance use treatment program in the Midwestern United States where individuals could opt to live in structured sober living during outpatient treatment [174]. Living in recovery housing was associated with greater likelihood of satisfactory discharge and longer length of stays in outpatient treatment when compared with a group living elsewhere.

More recently, researchers have tested the effectiveness of different ways of offering recovery housing, including providing abstinent contingencies. Tuten and colleagues [175] randomized people who completed medication‐assisted opioid detoxification to one of three groups: recovery housing alone (RH), abstinent‐contingent recovery housing plus reinforcement‐based therapy (RH + RBT) and usual care (UC). The recovery housing was paid for by the study during the 12‐week protocol contingent on participant abstinence from opioids and cocaine as determined by on‐site testing at a separate treatment program. Overall rates of drug abstinence were 50% for RH + RBT, 37% for RH and 13% for UC [175]. A second quasi‐experimental design showed that individuals who accessed recovery housing, irrespective of whether it was provided as part of the intervention (RH + RBT) or obtained on their own (RBT without recovery housing), had better abstinence and employment outcomes than those not accessing recovery housing [176].

### Policy and practice implications

Recovery housing shows promising results in well designed and executed studies in the United States. However, the housing market, healthcare and social benefit systems are markedly different in England, and it is not clear that these results are directly translatable. The UK government defines ‘supported housing’ as ‘accommodation provided alongside support, supervision or care to help people live as independently as possible in the community’, and people recovering from AOD disorder are just one group who may benefit from this system [177]. There are efforts underway to develop a comprehensive listing of recovery residences in the United States [178], and a similar exercise is urgently required in England. Better definition of different types of recovery housing combined with minimum quality standards would help attempts to evaluate their effectiveness, cost effectiveness and could support their wider provision. Any future development in UK recovery housing should also ensure that provision is directly linked to the wider ROSC in each locality.

## CONTINUING CARE AND RECOVERY CHECK‐UPS

Between half and two‐thirds of people receiving treatment for severe AOD use disorders relapse within a year of entering treatment, and the risk of relapse remains high throughout the early years of recovery [179, 180, 181, 182, 183]. Individuals often seek multiple episodes of treatment before achieving sustained remission from problem AOD use and may cycle through periods of short‐term abstinence, relapse and treatment over several years. Early readmission to treatment after relapse is associated with an increased likelihood of achieving sustained abstinence and a reduced likelihood of mortality [184]. An episode of treatment may be followed by ‘aftercare’, which is the delivery of interventions that aim to sustain symptom remission (e.g. abstinence), usually of a lower degree of intensity, and often delivered in non‐clinical or peer‐led settings. A ROSC views these post‐treatment activities as essential and renames them ‘continuing care’ [36], a period of less intensive treatment and recovery support following a more intensive initial treatment episode. Clinical models of long‐term recovery management have been extended in duration over time (‘extensive’ rather than ‘intensive’ care) and have started to use new communication technologies to supplement face‐to‐face contact [185].

Systematic reviews have explored the evidence for continuing care interventions or longer‐term recovery management strategies [30, 36, 186, 187, 188], and have included RCTs, quasi‐experimental and single group prospective studies. The earliest review included studies of continuing care compared with no or minimal continuing care, as well as studies comparing two or more active continuing care interventions [187]. Approximately half of the interventions produced positive effects, and the most effective continuing care interventions were of at least 12 months planned duration and involved more active efforts to engage and retain participants [187]. A subsequent meta‐analysis focused on 19 RCTs published (up until 2010) that compared continuing care for AOD use disorders with minimal or no continuing care [186]. The authors found a small but significant benefit for continuing care on AOD outcomes at the end of the interventions and at posttreatment follow‐up. Later reviews have divided the interventions into continuing care strategies and ‘long‐term recovery management’ (RM) strategies, organising the former as a function of intervention modality: (1) continuing care delivered face‐to‐face; (2) continuing care delivered by telephone; and (3) continuing care delivered by digital platform.

### Face‐to‐face continuing care

Face‐to‐face continuing care (F2F‐CC) interventions include early warning signs of relapse prevention training [189], behavioural contracting [190, 191, 192], mindfulness‐based relapse prevention [193], coping skills training [194], individual or group cognitive‐behavioural relapse prevention training [195, 196] or behavioural marital therapy [197]. These approaches vary in intensity, duration and structure, involving eight to 26 sessions over 3 to 6 months and follow a more intensive period of treatment [36]. Reviews of the RCTs suggest that such interventions promote modest, but inconsistent benefit on AOD outcomes compared to care as usual. However, usual care typically consisted of a group program that encouraged 12‐step group attendance while supporting individuals to cope with recovery‐related challenges, and such potent and active comparators often produced outcomes that were as good, or better, than the continuing care intervention itself.

### Telephone‐based continuing care

Telephone‐based continuing care (T‐CC) interventions consist of telephone‐based monitoring and/or counselling lasting 5 to 30 minutes in addition to continuing care of varying intensity [36, 188]. The telephone calls continue for between 12 weeks and 12 months, usually tapering down in frequency from weekly to monthly over this period. T‐CC for alcohol use disorders (AUD) appears to be at least as effective as F2F‐CC, with small to moderate benefit with some exceptions [188]. These interventions work best where there is a defined mechanism of change, and any benefit appears to dissipate once the intervention ends. The findings for individuals with drug use disorders are more varied, with some studies showing no greater benefit for T‐CC (or even negative effects) and others yielding positive effects in the full sample or in higher‐risk subsamples [188]. Women with a primary cocaine use disorder may benefit from telephone‐based interventions more than men [198].

Despite the modest benefit in terms of outcomes, T‐CC is cost‐effective relative to usual care, ultimately reducing the total cost to society and the individual by an additional $750 to $800 per individual per year [199, 200]. Brief telephone‐based interventions appear to be less effective than face‐to‐face interventions for individuals with greater clinical severity. For example, an evaluation of a standard 12‐week telephone monitoring and counselling protocol in individuals receiving inpatient psychiatric treatment for co‐occurring SUD and a psychiatric disorder found that the telephone‐based intervention did not improve substance use outcomes or increase attendance at self‐help programs compared to standard care [201]. Individuals with more network support for drinking, low readiness to change and prior treatment at the start of the intervention may benefit most from intensive T‐CC interventions delivered over extended periods (e.g. 2 years) [202].

### Digital technology‐assisted delivery of continuing care

Digital technology may be used in a variety of ways to deliver continuing care interventions [188, 203]. Smartphone applications (apps) and texting programs can be used to augment behavioural interventions by providing automated support between usual therapy sessions and to convey information on progress back to the therapist. Technological modes of continuing care delivery may provide a menu of features designed to support recovery (e.g. self‐monitoring, managing high‐risk situations, tools for relaxation or distraction and connection with peers). One example is the smartphone‐based Addiction‐Comprehensive Health Enhancement Support System (A‐CHESS) application that provides easy access to relapse prevention resources alongside Global Positioning System‐based ‘geo‐fencing’, a system enabling the individual to avoid areas that induce craving and trigger relapse. Supplementing usual care with the A‐CHESS app increased the odds of abstinence from alcohol 12 months after residential treatment by 65%, in addition to a smaller reduction in non‐heavy drinking days [204]. These results appear to be mediated by increased participation in outpatient treatment rather than increased attendance at mutual‐help groups [205]. A second continuing care trial for people with AUD found providing a smartphone with a data plan and A‐CHESS for 12 months significantly reduced days of alcohol use and heavy alcohol use over that period relative to people who did not receive A‐CHESS [188].

Text message‐based digital technology‐assisted delivery of continuing care (D‐CC) interventions can monitor self‐selected drinking goals and provide motivational text messages and telephone calls when participants fail to achieve goals or ask for support [206]. Compared to standard continuing care, such an intervention has been shown to reduce the rate of at‐risk drinking from 42% to 29%, and participants responded to 88% of the SMS prompts in the D‐CC condition. Another RCT tested the efficacy of a novel, fully automated D‐CC intervention known as alcohol therapeutic interactive voice response (ATIVR) [207]. Participants called into the interactive voice response (IVR) system once per day to report on factors such as substance use, mood, craving, self‐efficacy, risky situations, substance‐free recreation and coping. Based on this information, tailored feedback was provided if the individual was judged to be at risk of lapse. Individuals with AUD who had completed 12 weeks of cognitive behavioural therapy were randomized to 4 months of the IVR system or usual care, and followed for 12 months [207]. Most primary analyses indicated no differences in drinking outcomes between the two conditions.

### Long‐term recovery management: recovery management check‐ups

Recovery management check‐ups (RMC) are a form of continuing care intervention that provide individuals who have entered treatment for AOD disorder with long‐term monitoring and active attempts to reengage them in treatment when needed [208, 209]. In RMC, the person receiving support is assessed face‐to‐face every 3 months using urine drug testing and standardized questionnaires. If the clinical assessment indicates a need for active treatment, a dedicated ‘linkage manager’ uses motivational interviewing techniques to help them recognize and acknowledge their resumption of AOD use and discuss the need for additional treatment. Formal barriers to re‐entering treatment are addressed (e.g. by organising transport).

Three RCTs of the RMC intervention have found modest, but reliable benefit compared to usual care/assessment‐only across a range of recovery outcomes [209, 210]. These include a reduction in use and use‐related problems and reduced time to treatment when needed [208, 211]. This approach is cost‐effective, incurring similar societal and intervention‐related costs compared to assessment without intervention over time, but producing better outcomes [199]. As with other continuing care models, individuals with more complex needs (e.g. history of criminal justice involvement and substance onset before age 15) may derive the most benefit from RMCs [208].

### Policy and practice implications

If AOD use disorder is conceptualised as a chronic condition, continuing care should be recognised as an important component of a ROSC. Reviews report small to moderate beneficial effects when results from individual studies are averaged or combined. However, there is some evidence that continuing care of longer duration that includes more active efforts to keep people engaged may produce more consistently positive results. Moreover, people at higher risk for relapse by virtue of continued substance use in the first phase of care, or poor social support or low motivation early in treatment, may benefit to a greater degree from continuing care than those with a better prognosis [198, 212].

The research in this area is exclusively conducted in the United States where the length of a treatment intervention may be dictated by an insurance plan. In the United Kingdom, community‐based treatment services tend to be free at the point of delivery and rarely have a fixed length. Therefore, continuing care interventions may be delivered further into a treatment career, and digital interventions may be more appealing. Although digital interventions have produced some positive effects on AOD use outcomes, significant challenges remain in providing D‐CC using smartphone apps and text messaging. Potential users may not have access to a smartphone and/or data plan or a mobile phone for SMS‐based interventions [213], and geo‐location may be treated with suspicion [213].

Recovery check‐ups have been included as an intervention reported to the English national drug treatment monitoring system (NDTMS) for 8 years [214]. However, there is evidence from a 2022 survey of local authority addiction treatment commissioners in England have limited understanding of what recovery check‐ups are [27]. Continuing care is even less familiar to UK stakeholders. The NDTMS definition of a recovery check‐up has recently been refined to bring it closer into line with evidence. Continuing care has also been added to the NDTMS dataset as a ‘placeholder’ should future delivery become integral to the English service delivery model, but there is no current requirement for this to be completed. This reflects the status of this intervention as one difficult to translate into an English context and requiring further consideration of its potential role within treatment and recovery service models.

## RECOVERY COMMUNITY CENTRES

Described as the ‘new kids on the block’, recovery community centres (RCCs) were first acknowledged in the United States in the early 2000s [215]. They provide a ‘one‐stop shop’ for RSS including recovery coaching, relapse prevention skills‐building, support for employment and training, recreational activities and a range of other support services intended for people seeking recovery [215]. They may be run by any combination of peer volunteers, peer support workers and/or addiction treatment staff and support all pathways to recovery by not promoting any specific philosophy or model (e.g. 12‐step or religious) [215, 216]. RCCs are designed to help people build their recovery capital, generate meaning and purpose, derive a positive social identity and engage with a positive peer group. A similar novel type of recovery community support service couples peer‐based recovery support with physical exercise and recreational activities. The best known US example of these is The Phoenix (www.thephoenix.org), which provides on‐site workout classes (e.g. rock climbing, weightlifting, cross‐fit and yoga) via a network of sober gyms, as well as organizing outings and other recreational activities.

Early single site, prospective evaluations of RCCs in Pennsylvania, New York and Oregon found improvements at 6‐month follow‐up in participant substance use, employment, education, housing and criminal justice status [217, 218, 219]. More recently a prospective cohort study of 32 RCCs across New England and New York State incorporated on‐site ratings of the physical environment, director interviews and staff and participant surveys, as well as documenting outcomes in new participants. On average, they were accessed by several hundred people per month, serving as ‘community hubs’ in dense urban populations for typically low‐income, white, unemployed men who had a history of a primary opioid (41%) or alcohol (38%) problems and lifetime psychiatric comorbidity. Most participants were in the early stages of recovery stabilization and had been involved with the criminal justice system, but a proportion was in longer‐term recovery of several years. Most (84%) of the paid staff and volunteers were in recovery [215]. RCCs provided a range of services including social/recreational (100%), recovery coaching (77%), technology/internet (76%), employment (83%) and education (63%) assistance. Medication‐assisted treatment support (43%) and overdose reversal training (57%) were also offered and rated highly important by staff.

Statistical modelling on these data showed that greater RCC exposure (as measured by number of years of RCC involvement, percentage days of RCC attendance in past 90 days and length of typical RCC visit) was associated with greater accrual of recovery capital, but not social support [216]. However, both recovery capital and greater social support were associated with theorized higher levels of QoL, self‐esteem and lower psychological distress [216]. A smaller prospective, subgroup study of 275 new starters at a single RCC found participants had a mean age of 38.7 years, were racially diverse, single, unemployed and poorly educated, with a history of psychiatric problems, low QoL and prior treatment/mutual help participation [220]. Individuals attended 1 to 2 times/week on average, with greater RCC engagement at 3 months predicted by Hispanic ethnicity, shorter travel time to the RCC (<15 minutes), prior treatment, lower initial social support and relatively greater baseline QoL. In longitudinal analyses, RCC involvement was associated with improvements in duration of abstinence, AOD problems, psychological well‐being and QoL, but not in recovery assets.

### Policy and practice implications

RCCs seem to fulfil a role not provided either by formal treatment or by mutual help groups in building recovery capital in the long term and thereby enhancing functioning and QoL [216]. RCCs may play a valuable role for those suffering from primary opioid problems who tend to be in need of more services [221], feel more stigmatized [222] and have been shown to have lower recovery capital and QoL compared to those with primary alcohol problems in early recovery [223]. New models combining OAT and RCCs have been described in the United States [68], and RCCs appear to engage and provide benefits for individuals with most problems, the lowest QoL and the fewest resources [220]. The provision of physical health‐promoting activities in an openly supportive, explicitly recovery‐friendly environment provides another type of recovery pathway that can attract and engage individuals with AOD disorders. This strengths‐based, resilience‐focused combination of physical activities and social networks may be particularly attractive to young people in helping them to sustain remission and build recovery capital.

United Kingdom guidance promotes the development of RCCs through the growth of LEROs [27] and ‘recovery cafés’, which are the closest approximation to the RCC model described above [224]. The 2021 strategy ‘From Harm to Hope’ advocates joint working by healthcare and social care providers, peer‐led organisations and local government to create recovery hubs in each locality in England [32]. However, detailed descriptions of the operation of such services, markers of quality in terms of their delivery and sustainable funding structures are required to develop an evidence‐base relevant to England.

## RSS IN EDUCATIONAL SETTINGS

### Collegiate recovery programs

Engagement in peer‐supported substance‐free environments has been associated with reduced substance use and increased psychosocial functioning among young people [225, 226]. Tertiary education‐based RSS began in the United States in the late 1970s, offering drug‐free housing options, individual or group counselling to work on recovery and academic issues, relapse prevention and ‘life skills’ training and sober leisure activities. The emphasis was on peer support and the 12‐step program, supported by a small core of paid staff. Early evidence for their success in promoting recovery and preventing relapse [227] led to the award of federal funding to Texas Tech University in 2005 to document the key elements of its RSS on campus. Their ‘Collegiate Recovery Program’ (CRP) model was designed to inform other universities about how to implement RSS on campus. A rapid increase in number of CRPs in the United States followed, and estimates from the Association for Recovery in Higher Education put the number at approximately 150 established or developing CRPs in 2020 [228].

A systematic review reported 18 non‐controlled studies describing the development of CRPs between 1988 and 2017 [228]. Early studies at Texas Tech examined the student experience and the mechanisms by which they remained resilient within high‐risk environments [227, 229]. The first nationwide demographic survey of CRP students included 429 respondents from 29 CRPs, mostly White males with a mean age of 26. A third had experienced an episode of homelessness, over half had been arrested and 70% had co‐occurring mental health problems. Most expressed a desire or need for a supportive peer network, and over a third felt that they would not be at university without the CRP [230, 231].

A scoping review of research on services for students in addiction recovery identified 54 studies [232]. Clinical outcomes (35%) and recovery experiences (28%) of university students were most often examined, and two‐thirds were published after 2015. Studies examining clinical outcomes looked specifically at substance use, and the majority were cross‐sectional observational designs including current university students in recovery. Reported rates of return to substance use varied, including 2.2% of current CRP students at a Midwestern university who had returned to harmful use of alcohol or drugs after 6 months in the program [233], a 4.4% student return to use rate (within‐semester) at a university in Texas [229] and a 10.2% return to use rate in CRP alumni since graduation in a national sample [234]. Other reported outcomes have included reduced craving [235, 236], increased recovery‐related social support [237], improved coping with temptations [238], less disordered eating [239] and improvements in multiple medical/mental health conditions [240].

Studies exploring recovery experience have often used qualitative research methodology to capture and interpret student descriptions of their lived experiences [226, 241, 242, 243]. A meta‐synthesis of studies evaluating the impact of CRPs using qualitative analysis reported six ‘metaphors’ that were central to their activity [244]: (1) social connectivity, an important benefit of a CRP is a ready‐made group of people who have direct experience of the potential issues facing the student in recovery [231, 245]; (2) recovery support, including timetabled events and services that accommodate recovery needs within a CRP [231, 246, 247, 248]; (3) drop‐in recovery centres are a dedicated space on campus supportive of recovery that provide a buffer to the dominant narrative of ‘partying’, providing support to cope with the stress of the transition to university life; (4) internalized feelings, including identity, values, coherence and development [244] (overcoming stigma, shame and exclusion is a major part of this journey) [245, 248]; (5) coping mechanisms, including dealing with stress, resolving conflicts, improving emotional regulation and other cognitive behavioural changes; and (6) conflict of recovery/student status, which is success for individuals in recovery that includes participation in ever‐widening social circles while navigating value conflicts in pro‐social ways. The particularly challenging areas are intimate relationships, dating and social activities [241].

### Recovery high schools

The process of supporting recovery in young people moved further upstream in the United States in the 1980s with the development of recovery high schools (RHS) [249]. The underpinning principle behind them is the same as the CRP, namely that schools are crucial social environments for young people in recovery from AOD use disorders. Some standalone RHS exist, but many are embedded within another school [250]. Pupil numbers range from two to 115, and unlike CRPs the emphasis is on staff‐ rather than peer‐leadership [251]. Although no single RHS model exists, education‐based RSS have continued to grow in recent years, with a reported 40 RHS currently in operation [252].

Two cross‐sectional studies provided the earliest information on the RHS [250, 253]. Moberg and Finch [254] surveyed 321 students across 17 RHS in six states and found that 78% of participants reported past addiction treatment, and 80% currently attended weekly 12‐step fellowship meetings. The proportion of students reporting at least weekly use of alcohol, cannabis or other illicit drugs fell from 90% in the 12‐month period before entering the school to 7% while attending. Participants who had attended the school for at least 90 days (*n* = 174) retrospectively reported an average 32% of days abstinent (PDA) from substances in the 90 days before starting, compared with an average PDA of 82% since they had started at the school.

A systematic review [255] published in 2018 found just one study that met inclusion criteria. The study recruited 293 adolescents and their caregiver(s) in three states (Minnesota, Wisconsin and Texas) and followed them longitudinally over a 12‐month period. At the 6‐month follow‐up point the authors compared outcomes for students who self‐selected to attend a RHS with a comparison group of students who did not enrol in a RHS [250]. Reviewers concluded that RHS may reduce students' school absenteeism, cannabis and other drug use and increase abstinence from drugs. However, they may also be no better or worse than other high schools in improving grades, reducing truancy or reducing alcohol use [255].

### Policy and practice implications

CRPs on US university campuses are increasing in both number and size, and the number of evaluation reports in the academic literature is growing steadily [232]. In contrast, there is relatively little good data on AOD use in UK university student populations [256] or indeed young people generally [257]. The first university‐led CRP has been described in England [258] and the model shows promise as a relatively cost‐effective strategy for raising awareness of the concepts of addiction and recovery. Theoretical evaluation frameworks have been proposed [259, 260], and prospective data collection is underway in the United States [261]. However, until sufficient pilot sites are developed it will be difficult to collect data to support effectiveness and cost‐effectiveness in England.

## RECOMMENDATIONS AND CONCLUSIONS

This monograph provides a structure to help policy makers, commissioners and service providers describe and understand the emerging field of RSS, which has seen a consensus about its components developing among commentators in the past decade [34, 35, 44, 262]. The six forms of RSS described here vary in the degree to which ‘lived experience’ is a key aspect (high for P‐BRSS, low for employment or continuing care/RMC interventions) and in whether delivery occurs in existing treatment spaces (e.g. continuing care/RMS, or some P‐BRSS interventions) or in the community (housing, employment, educational and RCC interventions). Although there is a large overlap between many of the components of the interventions described here, having a common language to describe what can be delivered may help in conceptualising the ROSC with more clarity.

In a 2009 white paper by the US Department of Health exploring research supporting the ROSC, Sheedy and Whitter [263] concluded that ‘while many of the principles and systems elements are easily supported by existing literature in the addictions field, research supporting others was more difficult to find. In some circumstances, they were supported by literature outside of addictions research, primarily through the mental health and public health research fields’. The focus of this monograph was to explore the breadth and depth of the supporting evidence base for RSS in the addictions field. Some of the six forms of RSS can be said to be well supported, with RCTs of interventions to increase levels of employment demonstrating large effect sizes. Likewise, continuing care interventions that aim to extend treatment intervention into the early recovery phase have shown small but significant benefit in several RCTs. Peer‐delivered interventions to link people in active addiction, and those in remission, to ongoing support are associated with decreased rates of relapse and re‐admission, increased engagement and increased social support for change. However, the variability in the design of these studies, the definition of peer support and the participating population, mean that further work is required to be clear about the effective components of the intervention. Studies of recovery housing have also shown positive results, including significant differences from standard care as well as early efforts to delineate the mechanisms underpinning these findings. No controlled studies exist to support RCCs or RSS in educational settings, but the complexity of these interventions and the wide range of potential outcome measures mean that other study designs may be more relevant. In sum, a considerable body of evidence to support the recovery support service components of the ROSC in the addictions field has emerged since the report by Sheedy and Whitter [263].

A challenge facing policy makers is how to evaluate the ROSC as a whole. Although the evidence presented in this monograph has delineated some of the component parts of a recovery‐oriented system, there is no easy way of measuring the benefit or otherwise of adopting this approach in a locality. White has described an array of recovery‐focused system performance measures, including indicators of infrastructure and adaptive capacity, recovery‐focused service process measures and recovery outcome measures [264]. If data collection ends when the individual leaves the treatment setting it will be hard to demonstrate the impact of interventions designed to build recovery in the community. The concept of recovery capital provides a useful shorthand for the overall goal of RSSs [24], and several research instruments are now available to measure it [265]. Measures of recovery capital can be incorporated into individual goal planning strategies, using smartphone‐based feedback to guide progress [266].

Policy makers in England have begun to strengthen the mechanisms for increased and sustained provision of RSS within the existing treatment system. A ‘Commissioning Quality Standard’ issued in 2022 included ‘Recovery‐focused support’ (including housing, learning and employment, personal finance, healthcare, social connectivity, meaningful activity and mutual aid) in a section entitled ‘Providing a full range of evidence‐based support’ [267]. Each local treatment system is expected to address these areas to access new funding streams. The process was supported by national guidance explaining the importance of lived experience and the need for peer‐led services to complement services provided by treatment providers [27]. This has presented an opportunity to define some of the interventions described (e.g. recovery housing), which in turn will allow systems to start collecting routine data to evaluate efficacy. It will also be important to ensure that definitions of the ‘peer support worker’ role and any required competencies, training and supervision to deliver it are understood and translate into practice. This will ensure that the individual delivering peer support is both effective and supported to sustain their own recovery journey.

In conclusion, chronic AOD use problems are considered high volume, high burden disorders in most middle‐ and high‐income countries. The attributable economic cost associated with lost productivity, criminal justice involvement and increased health care utilization is immense and growing, running into trillions of dollars worldwide. The growing range of RSS described here are proving to have not only clinical and public health utility, but also cost utility, with several studies now showing their potential to reduce the healthcare, as well as broader societal, cost burden. A rational social and fiscal response to endemic AOD challenges should, therefore, include the more intensive acute care clinical services linked with more extensive community‐based RSSs. Further investigation is needed in England to understand more about who may need which type of service, when, for what duration and at what intensity. However, results outlined here suggest distinct clinical, public health, social and fiscal advantages to developing and implementing RSSs as key components of a comprehensive, effective ROSC.

## AUTHOR CONTRIBUTIONS

**Ed Day**: Conceptualisation; methodology; project administration; writing—original draft; writing—review and editing. **Laura Charlotte Pechey**: Data curation; writing—review and editing. **Suzie Roscoe**: Writing—review and editing. **John F. Kelly**: Conceptualisation; methodology; writing—original draft; writing—review and editing.

## DECLARATION OF INTERESTS

None.

## Supporting information


**Data S1** Supporting Information
