# Supplementary material for: Recovery support services as part of the continuum of care for alcohol or drug use disorders
Source: Addiction. 2025 Jan 28;120(8):1497–520. doi: 10.1111/add.16751 (PMC12215296; doi:10.1111/add.16751)
Supplement: Supplementary file 1 — Data S1 Supporting Information [file ADD-120-1497-s001.docx]

**Search methods used by original systematic review by Kelly et al. (2017) – Systematic Review Search Syntax**

***Inclusion/Exclusion Criteria***

1. Quantitative Data

2. Measured substance use outcome (abstinence, drinking intensity, consequences), other marker of SUD recovery (quality of life, psychiatric symptoms, etc.), and/or health care costs

3. Adolescent or adult – no limitations on age range

4. No coerced populations or studies where individuals are institutionalized while receiving the recovery support service (e.g., residential treatment, in jail/prison)

5. Use a hierarchy for research design. Only include second tier if no first tier are available

Tier 1: Use of a comparison group measuring outcomes over time (e.g., recovery support service vs. no recovery support service), including RCTs and quasi-experimental (e.g., comparison of two naturally formed groups)

Tier 2: Single group pre-post prospective or retrospective cross-sectional designs, other cross-sectional designs (note: if longitudinal, but involvement in recovery support service is measured at baseline as predictor of SUD outcome, like abstinence, this is considered cross-section, i.e., in Tier 2)

**A. Peer-based recovery support services**

**Pubmed**

((“Recovery coaching”[Title/Abstract] OR “Peer recovery support ”[Title/Abstract] OR “Peer-based recovery support services”[Title/Abstract] OR “Individual peer support”[Title/Abstract])) AND ((recovery[Title/Abstract] OR remission[Title/Abstract] OR abstinence[Title/Abstract] OR "harm reduction"[Title/Abstract] OR “substance abuse”[Title/Abstract] OR “substance misuse”[Title/Abstract] OR “substance dependence”[Title/Abstract] OR “drug dependence”[Title/Abstract] OR “substance use disorder”[Title/Abstract] OR “alcohol use disorder”[Title/Abstract] OR “drug use disorder”[Title/Abstract] OR alcohol*[Title/Abstract] OR marijuana[Title/Abstract] OR “THC”[Title/Abstract] OR cannabis[Title/Abstract] OR cocaine[Title/Abstract] OR heroin[Title/Abstract] OR opioid*[Title/Abstract] OR opiate*[Title/Abstract] OR narcotic*[Title/Abstract] OR amphetamine*[Title/Abstract] OR methamphetamine*[Title/Abstract] OR benzodiazepine*[Title/Abstract] OR barbiturate*[Title/Abstract] OR hallucinogen*[Title/Abstract] OR inhalant*[Title/Abstract] OR steroid*[Title/Abstract] OR “club drug*”[Title/Abstract] OR ecstasy[Title/Abstract] OR “MDMA”[Title/Abstract] OR stimulant*[Title/Abstract] OR cost-benefit[Title/Abstract] OR cost-offset[Title/Abstract] OR cost-effectiveness[Title/Abstract]) OR “cost benefit”[Title/Abstract] OR “cost offset”[Title/Abstract] OR “cost effectiveness”[Title/Abstract]))

**Embase**

(‘Recovery coaching’:ab,ti OR ‘Peer recovery support’:ab,ti OR ‘Peer-based recovery support services’:ab,ti OR ‘Individual peer support’:ab,ti) AND (recovery:ab,ti OR remission:ab,ti OR abstinence:ab,ti OR 'harm reduction':ab,ti OR ‘substance abuse’:ab,ti OR ‘substance misuse’:ab,ti OR ‘substance dependence’:ab,ti OR ‘drug dependence’:ab,ti OR ‘substance use disorder’:ab,ti OR ‘alcohol use disorder’:ab,ti OR ‘drug use disorder’:ab,ti OR alcohol*:ab,ti OR marijuana:ab,ti OR ‘THC’:ab,ti OR cannabis:ab,ti OR cocaine:ab,ti OR heroin:ab,ti OR opioid*:ab,ti OR opiate*:ab,ti OR narcotic*:ab,ti OR amphetamine*:ab,ti OR methamphetamine*:ab,ti OR benzodiazepine*:ab,ti OR barbiturate*:ab,ti OR hallucinogen*:ab,ti OR inhalant*:ab,ti OR steroid*:ab,ti OR ‘club drug*’:ab,ti OR ecstasy:ab,ti OR ‘MDMA’:ab,ti OR stimulant*:ab,ti OR cost-benefit:ab,ti OR cost-offset:ab,ti OR cost-effectiveness:ab,ti OR ‘cost benefit’:ab,ti OR ‘cost offset’:ab,ti OR ‘cost effectiveness’:ab,ti)

**CINAHL**

AB (“Recovery coaching” OR “Peer recovery support” OR “Peer-based recovery support services” OR “Individual peer support”) AND AB (recovery OR remission OR abstinence OR "harm reduction" OR “substance abuse” OR “substance misuse” OR “substance dependence” OR “drug dependence” OR “substance use disorder” OR “alcohol use disorder” OR “drug use disorder” OR alcohol* OR marijuana OR “THC” OR cannabis OR cocaine OR heroin OR opioid* OR opiate* OR narcotic* OR amphetamine* OR methamphetamine* OR benzodiazepine* OR barbiturate* OR hallucinogen* OR inhalant* OR steroid* OR “club drug*” OR ecstasy OR “MDMA” OR stimulant* OR cost-benefit OR cost-offset OR cost-effectiveness OR “cost benefit” OR “cost offset” OR “cost effectiveness”)

AB (“Recovery coaching” OR “Peer recovery support” OR “Peer-based recovery support services” OR “Individual peer support”) AND TI (recovery OR remission OR abstinence OR "harm reduction" OR “substance abuse” OR “substance misuse” OR “substance dependence” OR “drug dependence” OR “substance use disorder” OR “alcohol use disorder” OR “drug use disorder” OR alcohol* OR marijuana OR “THC” OR cannabis OR cocaine OR heroin OR opioid* OR opiate* OR narcotic* OR amphetamine* OR methamphetamine* OR benzodiazepine* OR barbiturate* OR hallucinogen* OR inhalant* OR steroid* OR “club drug*” OR ecstasy OR “MDMA” OR stimulant* OR cost-benefit OR cost-offset OR cost-effectiveness OR “cost benefit” OR “cost offset” OR “cost effectiveness”)

TI (“Recovery coaching” OR “Peer recovery support” OR “Peer-based recovery support services” OR “Individual peer support”) AND AB (recovery OR remission OR abstinence OR "harm reduction" OR “substance abuse” OR “substance misuse” OR “substance dependence” OR “drug dependence” OR “substance use disorder” OR “alcohol use disorder” OR “drug use disorder” OR alcohol* OR marijuana OR “THC” OR cannabis OR cocaine OR heroin OR opioid* OR opiate* OR narcotic* OR amphetamine* OR methamphetamine* OR benzodiazepine* OR barbiturate* OR hallucinogen* OR inhalant* OR steroid* OR “club drug*” OR ecstasy OR “MDMA” OR stimulant* OR cost-benefit OR cost-offset OR cost-effectiveness OR “cost benefit” OR “cost offset” OR “cost effectiveness”)

TI (“Recovery coaching” OR “Peer recovery support” OR “Peer-based recovery support services” OR “Individual peer support”) AND TI (recovery OR remission OR abstinence OR "harm reduction" OR “substance abuse” OR “substance misuse” OR “substance dependence” OR “drug dependence” OR “substance use disorder” OR “alcohol use disorder” OR “drug use disorder” OR alcohol* OR marijuana OR “THC” OR cannabis OR cocaine OR heroin OR opioid* OR opiate* OR narcotic* OR amphetamine* OR methamphetamine* OR benzodiazepine* OR barbiturate* OR hallucinogen* OR inhalant* OR steroid* OR “club drug*” OR ecstasy OR “MDMA” OR stimulant* OR cost-benefit OR cost-offset OR cost-effectiveness OR “cost benefit” OR “cost offset” OR “cost effectiveness”)

**CENTRAL (Cochrane Registry)**

Same as for CINAHL

**PsycINFO**

Same as for CINAHL

**B. Recovery community centers**

**Pubmed**

(("Recovery community center"[Title/Abstract] OR "Recovery center"[Title/Abstract] OR "Recovery support center"[Title/Abstract] OR "Peer support center"[Title/Abstract] OR "Recovery community organization"[Title/Abstract] OR "Peer participatory model"[Title/Abstract])) AND ((recovery[Title/Abstract] OR remission[Title/Abstract] OR abstinence[Title/Abstract] OR "harm reduction"[Title/Abstract] OR “substance abuse”[Title/Abstract] OR “substance misuse”[Title/Abstract] OR “substance dependence”[Title/Abstract] OR “drug dependence”[Title/Abstract] OR “substance use disorder”[Title/Abstract] OR “alcohol use disorder”[Title/Abstract] OR “drug use disorder”[Title/Abstract] OR alcohol*[Title/Abstract] OR marijuana[Title/Abstract] OR “THC”[Title/Abstract] OR cannabis[Title/Abstract] OR cocaine[Title/Abstract] OR heroin[Title/Abstract] OR opioid*[Title/Abstract] OR opiate*[Title/Abstract] OR narcotic*[Title/Abstract] OR amphetamine*[Title/Abstract] OR methamphetamine*[Title/Abstract] OR benzodiazepine*[Title/Abstract] OR barbiturate*[Title/Abstract] OR hallucinogen*[Title/Abstract] OR inhalant*[Title/Abstract] OR steroid*[Title/Abstract] OR “club drug*”[Title/Abstract] OR ecstasy[Title/Abstract] OR “MDMA”[Title/Abstract] OR stimulant*[Title/Abstract] OR cost-benefit[Title/Abstract] OR cost-offset[Title/Abstract] OR cost-effectiveness[Title/Abstract]) OR “cost benefit”[Title/Abstract] OR “cost offset”[Title/Abstract] OR “cost effectiveness”[Title/Abstract]))

**Embase**

('Recovery community center:ab,ti OR 'Recovery center:ab,ti OR 'Recovery support center:ab,ti OR 'Peer support center:ab,ti OR 'Recovery community organization:ab,ti OR 'Peer participatory model:ab,ti) AND (recovery:ab,ti OR remission:ab,ti OR abstinence:ab,ti OR 'harm reduction':ab,ti OR ‘substance abuse’:ab,ti OR ‘substance misuse’:ab,ti OR ‘substance dependence’:ab,ti OR ‘drug dependence’:ab,ti OR ‘substance use disorder’:ab,ti OR ‘alcohol use disorder’:ab,ti OR ‘drug use disorder’:ab,ti OR alcohol*:ab,ti OR marijuana:ab,ti OR ‘THC’:ab,ti OR cannabis:ab,ti OR cocaine:ab,ti OR heroin:ab,ti OR opioid*:ab,ti OR opiate*:ab,ti OR narcotic*:ab,ti OR amphetamine*:ab,ti OR methamphetamine*:ab,ti OR benzodiazepine*:ab,ti OR barbiturate*:ab,ti OR hallucinogen*:ab,ti OR inhalant*:ab,ti OR steroid*:ab,ti OR ‘club drug*’:ab,ti OR ecstasy:ab,ti OR ‘MDMA’:ab,ti OR stimulant*:ab,ti OR cost-benefit:ab,ti OR cost-offset:ab,ti OR cost-effectiveness:ab,ti OR ‘cost benefit’:ab,ti OR ‘cost offset’:ab,ti OR ‘cost effectiveness’:ab,ti)

**CINAHL**

AB ("Recovery community center" OR "Recovery center" OR "Recovery support center" OR "Peer support center" OR "Recovery community organization" OR "Peer participatory model") AND AB (recovery OR remission OR abstinence OR "harm reduction" OR “substance abuse” OR “substance misuse” OR “substance dependence” OR “drug dependence” OR “substance use disorder” OR “alcohol use disorder” OR “drug use disorder” OR alcohol* OR marijuana OR “THC” OR cannabis OR cocaine OR heroin OR opioid* OR opiate* OR narcotic* OR amphetamine* OR methamphetamine* OR benzodiazepine* OR barbiturate* OR hallucinogen* OR inhalant* OR steroid* OR “club drug*” OR ecstasy OR “MDMA” OR stimulant* OR cost-benefit OR cost-offset OR cost-effectiveness OR “cost benefit” OR “cost offset” OR “cost effectiveness”)

AB ("Recovery community center" OR "Recovery center" OR "Recovery support center" OR "Peer support center" OR "Recovery community organization" OR "Peer participatory model") AND TI (recovery OR remission OR abstinence OR "harm reduction" OR “substance abuse” OR “substance misuse” OR “substance dependence” OR “drug dependence” OR “substance use disorder” OR “alcohol use disorder” OR “drug use disorder” OR alcohol* OR marijuana OR “THC” OR cannabis OR cocaine OR heroin OR opioid* OR opiate* OR narcotic* OR amphetamine* OR methamphetamine* OR benzodiazepine* OR barbiturate* OR hallucinogen* OR inhalant* OR steroid* OR “club drug*” OR ecstasy OR “MDMA” OR stimulant* OR cost-benefit OR cost-offset OR cost-effectiveness OR “cost benefit” OR “cost offset” OR “cost effectiveness”)

TI ("Recovery community center" OR "Recovery center" OR "Recovery support center" OR "Peer support center" OR "Recovery community organization" OR "Peer participatory model") AND AB (recovery OR remission OR abstinence OR "harm reduction" OR “substance abuse” OR “substance misuse” OR “substance dependence” OR “drug dependence” OR “substance use disorder” OR “alcohol use disorder” OR “drug use disorder” OR alcohol* OR marijuana OR “THC” OR cannabis OR cocaine OR heroin OR opioid* OR opiate* OR narcotic* OR amphetamine* OR methamphetamine* OR benzodiazepine* OR barbiturate* OR hallucinogen* OR inhalant* OR steroid* OR “club drug*” OR ecstasy OR “MDMA” OR stimulant* OR cost-benefit OR cost-offset OR cost-effectiveness OR “cost benefit” OR “cost offset” OR “cost effectiveness”)

TI ("Recovery community center" OR "Recovery center" OR "Recovery support center" OR "Peer support center" OR "Recovery community organization" OR "Peer participatory model") AND TI (recovery OR remission OR abstinence OR "harm reduction" OR “substance abuse” OR “substance misuse” OR “substance dependence” OR “drug dependence” OR “substance use disorder” OR “alcohol use disorder” OR “drug use disorder” OR alcohol* OR marijuana OR “THC” OR cannabis OR cocaine OR heroin OR opioid* OR opiate* OR narcotic* OR amphetamine* OR methamphetamine* OR benzodiazepene* OR barbiturate* OR hallucinogen* OR inhalant* OR steroid* OR “club drug*” OR ecstasy OR “MDMA” OR stimulant* OR cost-benefit OR cost-offset OR cost-effectiveness OR “cost benefit” OR “cost offset” OR “cost effectiveness”)

**CENTRAL (Cochrane Registry)**

Same as for CINAHL

**PsycINFO**

Same as for CINAHL

**C. Recovery supports in educational settings**

**Pubmed**

((“collegiate recovery”[Title/Abstract] OR “recovery school”[Title/Abstract] OR “recovery high school”[Title/Abstract] OR “recovery hous*”[Title/Abstract] OR “university-based recovery center”[Title/Abstract] OR “university based recovery center”[Title/Abstract])) AND ((recovery[Title/Abstract] OR remission[Title/Abstract] OR abstinence[Title/Abstract] OR "harm reduction"[Title/Abstract] OR “substance abuse”[Title/Abstract] OR “substance misuse”[Title/Abstract] OR “substance dependence”[Title/Abstract] OR “drug dependence”[Title/Abstract] OR “substance use disorder”[Title/Abstract] OR “alcohol use disorder”[Title/Abstract] OR “drug use disorder”[Title/Abstract] OR alcohol*[Title/Abstract] OR marijuana[Title/Abstract] OR “THC”[Title/Abstract] OR cannabis[Title/Abstract] OR cocaine[Title/Abstract] OR heroin[Title/Abstract] OR opioid*[Title/Abstract] OR opiate*[Title/Abstract] OR narcotic*[Title/Abstract] OR amphetamine*[Title/Abstract] OR methamphetamine*[Title/Abstract] OR benzodiazepine*[Title/Abstract] OR barbiturate*[Title/Abstract] OR hallucinogen*[Title/Abstract] OR inhalant*[Title/Abstract] OR steroid*[Title/Abstract] OR “club drug*”[Title/Abstract] OR ecstasy[Title/Abstract] OR “MDMA”[Title/Abstract] OR stimulant*[Title/Abstract] OR cost-benefit[Title/Abstract] OR cost-offset[Title/Abstract] OR cost-effectiveness[Title/Abstract]) OR “cost benefit”[Title/Abstract] OR “cost offset”[Title/Abstract] OR “cost effectiveness”[Title/Abstract]))

**Embase**

(‘collegiate recovery’:ab,ti OR ‘recovery school’:ab,ti OR ‘recovery high school’:ab,ti OR ‘recovery hous*’:ab,ti OR ‘university-based recovery center’:ab,ti OR ‘university based recovery center’:ab,ti) AND (recovery:ab,ti OR remission:ab,ti OR abstinence:ab,ti OR 'harm reduction':ab,ti OR ‘substance abuse’:ab,ti OR ‘substance misuse’:ab,ti OR ‘substance dependence’:ab,ti OR ‘drug dependence’:ab,ti OR ‘substance use disorder’:ab,ti OR ‘alcohol use disorder’:ab,ti OR ‘drug use disorder’:ab,ti OR alcohol*:ab,ti OR marijuana:ab,ti OR ‘THC’:ab,ti OR cannabis:ab,ti OR cocaine:ab,ti OR heroin:ab,ti OR opioid*:ab,ti OR opiate*:ab,ti OR narcotic*:ab,ti OR amphetamine*:ab,ti OR methamphetamine*:ab,ti OR benzodiazepine*:ab,ti OR barbiturate*:ab,ti OR hallucinogen*:ab,ti OR inhalant*:ab,ti OR steroid*:ab,ti OR ‘club drug*’:ab,ti OR ecstasy:ab,ti OR ‘MDMA’:ab,ti OR stimulant*:ab,ti OR cost-benefit:ab,ti OR cost-offset:ab,ti OR cost-effectiveness:ab,ti OR ‘cost benefit’:ab,ti OR ‘cost offset’:ab,ti OR ‘cost effectiveness’:ab,ti)

**CINAHL**

AB (“collegiate recovery” OR “recovery school” OR “recovery high school” OR “recovery hous*” OR “university-based recovery center” OR “university based recovery center”) AND AB (recovery OR remission OR abstinence OR "harm reduction" OR “substance abuse” OR “substance misuse” OR “substance dependence” OR “drug dependence” OR “substance use disorder” OR “alcohol use disorder” OR “drug use disorder” OR alcohol* OR marijuana OR “THC” OR cannabis OR cocaine OR heroin OR opioid* OR opiate* OR narcotic* OR amphetamine* OR methamphetamine* OR benzodiazepine* OR barbiturate* OR hallucinogen* OR inhalant* OR steroid* OR “club drug*” OR ecstasy OR “MDMA” OR stimulant* OR cost-benefit OR cost-offset OR cost-effectiveness OR “cost benefit” OR “cost offset” OR “cost effectiveness”)

AB (“collegiate recovery” OR “recovery school” OR “recovery high school” OR “recovery hous*” OR “university-based recovery center” OR “university based recovery center”) AND TI (recovery OR remission OR abstinence OR "harm reduction" OR “substance abuse” OR “substance misuse” OR “substance dependence” OR “drug dependence” OR “substance use disorder” OR “alcohol use disorder” OR “drug use disorder” OR alcohol* OR marijuana OR “THC” OR cannabis OR cocaine OR heroin OR opioid* OR opiate* OR narcotic* OR amphetamine* OR methamphetamine* OR benzodiazepine* OR barbiturate* OR hallucinogen* OR inhalant* OR steroid* OR “club drug*” OR ecstasy OR “MDMA” OR stimulant* OR cost-benefit OR cost-offset OR cost-effectiveness OR “cost benefit” OR “cost offset” OR “cost effectiveness”)

TI (“collegiate recovery” OR “recovery school” OR “recovery high school” OR “recovery hous*” OR “university-based recovery center” OR “university based recovery center”) AND AB (recovery OR remission OR abstinence OR "harm reduction" OR “substance abuse” OR “substance misuse” OR “substance dependence” OR “drug dependence” OR “substance use disorder” OR “alcohol use disorder” OR “drug use disorder” OR alcohol* OR marijuana OR “THC” OR cannabis OR cocaine OR heroin OR opioid* OR opiate* OR narcotic* OR amphetamine* OR methamphetamine* OR benzodiazepine* OR barbiturate* OR hallucinogen* OR inhalant* OR steroid* OR “club drug*” OR ecstasy OR “MDMA” OR stimulant* OR cost-benefit OR cost-offset OR cost-effectiveness OR “cost benefit” OR “cost offset” OR “cost effectiveness”)

TI (“collegiate recovery” OR “recovery school” OR “recovery high school” OR “recovery hous*” OR “university-based recovery center” OR “university based recovery center”) AND TI (recovery OR remission OR abstinence OR "harm reduction" OR “substance abuse” OR “substance misuse” OR “substance dependence” OR “drug dependence” OR “substance use disorder” OR “alcohol use disorder” OR “drug use disorder” OR alcohol* OR marijuana OR “THC” OR cannabis OR cocaine OR heroin OR opioid* OR opiate* OR narcotic* OR amphetamine* OR methamphetamine* OR benzodiazepine* OR barbiturate* OR hallucinogen* OR inhalant* OR steroid* OR “club drug*” OR ecstasy OR “MDMA” OR stimulant* OR cost-benefit OR cost-offset OR cost-effectiveness OR “cost benefit” OR “cost offset” OR “cost effectiveness”)

**CENTRAL (Cochrane Registry)**

Same as for CINAHL

**PsycINFO**

Same as for CINAHL

**E. Recovery Housing**

**Pubmed**

((“oxford house”[Title/Abstract] OR “oxford home”[Title/Abstract] OR “sober living”[Title/Abstract] OR “sober living ho*”[Title/Abstract] OR “sober living environment”[Title/Abstract] OR “recovery residence”[Title/Abstract] OR “halfway house”[Title/Abstract] OR “halfway residence”[Title/Abstract] OR “transitional house”[Title/Abstract] OR domiciliary[Title/Abstract] OR “wet house”[Title/Abstract] OR “dry house”[Title/Abstract])) AND ((recovery[Title/Abstract] OR remission[Title/Abstract] OR abstinence[Title/Abstract] OR "harm reduction"[Title/Abstract] OR “substance abuse”[Title/Abstract] OR “substance misuse”[Title/Abstract] OR “substance dependence”[Title/Abstract] OR “drug dependence”[Title/Abstract] OR “substance use disorder”[Title/Abstract] OR “alcohol use disorder”[Title/Abstract] OR “drug use disorder”[Title/Abstract] OR alcohol*[Title/Abstract] OR marijuana[Title/Abstract] OR “THC”[Title/Abstract] OR cannabis[Title/Abstract] OR cocaine[Title/Abstract] OR heroin[Title/Abstract] OR opioid*[Title/Abstract] OR opiate*[Title/Abstract] OR narcotic*[Title/Abstract] OR amphetamine*[Title/Abstract] OR methamphetamine*[Title/Abstract] OR benzodiazepine*[Title/Abstract] OR barbiturate*[Title/Abstract] OR hallucinogen*[Title/Abstract] OR inhalant*[Title/Abstract] OR steroid*[Title/Abstract] OR “club drug*”[Title/Abstract] OR ecstasy[Title/Abstract] OR “MDMA”[Title/Abstract] OR stimulant*[Title/Abstract] OR cost-benefit[Title/Abstract] OR cost-offset[Title/Abstract] OR cost-effectiveness[Title/Abstract]) OR “cost benefit”[Title/Abstract] OR “cost offset”[Title/Abstract] OR “cost effectiveness”[Title/Abstract]))

**Embase**

(‘oxford house’:ab,ti OR ‘oxford home’:ab,ti OR ‘sober living’:ab,ti OR ‘sober living ho*’:ab,ti OR ‘sober living environment’:ab,ti OR ‘recovery residence’:ab,ti OR ‘halfway house’:ab,ti OR ‘halfway residence’:ab,ti OR ‘transitional house’:ab,ti OR domiciliary:ab,ti OR ‘wet house’:ab,ti OR ‘dry house’:ab,ti) AND (recovery:ab,ti OR remission:ab,ti OR abstinence:ab,ti OR 'harm reduction':ab,ti OR ‘substance abuse’:ab,ti OR ‘substance misuse’:ab,ti OR ‘substance dependence’:ab,ti OR ‘drug dependence’:ab,ti OR ‘substance use disorder’:ab,ti OR ‘alcohol use disorder’:ab,ti OR ‘drug use disorder’:ab,ti OR alcohol*:ab,ti OR marijuana:ab,ti OR ‘THC’:ab,ti OR cannabis:ab,ti OR cocaine:ab,ti OR heroin:ab,ti OR opioid*:ab,ti OR opiate*:ab,ti OR narcotic*:ab,ti OR amphetamine*:ab,ti OR methamphetamine*:ab,ti OR benzodiazepine*:ab,ti OR barbiturate*:ab,ti OR hallucinogen*:ab,ti OR inhalant*:ab,ti OR steroid*:ab,ti OR ‘club drug*’:ab,ti OR ecstasy:ab,ti OR ‘MDMA’:ab,ti OR stimulant*:ab,ti OR cost-benefit:ab,ti OR cost-offset:ab,ti OR cost-effectiveness:ab,ti OR ‘cost benefit’:ab,ti OR ‘cost offset’:ab,ti OR ‘cost effectiveness’:ab,ti)

**CINAHL**

AB (“oxford house” OR “oxford home” OR “sober living” OR “sober living ho*” OR “sober living environment” OR “recovery residence” OR “halfway house” OR “halfway residence” OR “transitional house” OR domiciliary OR “wet house” OR “dry house”) AND AB (recovery OR remission OR abstinence OR "harm reduction" OR “substance abuse” OR “substance misuse” OR “substance dependence” OR “drug dependence” OR “substance use disorder” OR “alcohol use disorder” OR “drug use disorder” OR alcohol* OR marijuana OR “THC” OR cannabis OR cocaine OR heroin OR opioid* OR opiate* OR narcotic* OR amphetamine* OR methamphetamine* OR benzodiazepine* OR barbiturate* OR hallucinogen* OR inhalant* OR steroid* OR “club drug*” OR ecstasy OR “MDMA” OR stimulant* OR cost-benefit OR cost-offset OR cost-effectiveness OR “cost benefit” OR “cost offset” OR “cost effectiveness”)

AB (“oxford house” OR “oxford home” OR “sober living” OR “sober living ho*” OR “sober living environment” OR “recovery residence” OR “halfway house” OR “halfway residence” OR “transitional house” OR domiciliary OR “wet house” OR “dry house”) AND TI (recovery OR remission OR abstinence OR "harm reduction" OR “substance abuse” OR “substance misuse” OR “substance dependence” OR “drug dependence” OR “substance use disorder” OR “alcohol use disorder” OR “drug use disorder” OR alcohol* OR marijuana OR “THC” OR cannabis OR cocaine OR heroin OR opioid* OR opiate* OR narcotic* OR amphetamine* OR methamphetamine* OR benzodiazepine* OR barbiturate* OR hallucinogen* OR inhalant* OR steroid* OR “club drug*” OR ecstasy OR “MDMA” OR stimulant* OR cost-benefit OR cost-offset OR cost-effectiveness OR “cost benefit” OR “cost offset” OR “cost effectiveness”)

TI (“oxford house” OR “oxford home” OR “sober living” OR “sober living ho*” OR “sober living environment” OR “recovery residence” OR “halfway house” OR “halfway residence” OR “transitional house” OR domiciliary OR “wet house” OR “dry house”) AND AB (recovery OR remission OR abstinence OR "harm reduction" OR “substance abuse” OR “substance misuse” OR “substance dependence” OR “drug dependence” OR “substance use disorder” OR “alcohol use disorder” OR “drug use disorder” OR alcohol* OR marijuana OR “THC” OR cannabis OR cocaine OR heroin OR opioid* OR opiate* OR narcotic* OR amphetamine* OR methamphetamine* OR benzodiazepine* OR barbiturate* OR hallucinogen* OR inhalant* OR steroid* OR “club drug*” OR ecstasy OR “MDMA” OR stimulant* OR cost-benefit OR cost-offset OR cost-effectiveness OR “cost benefit” OR “cost offset” OR “cost effectiveness”)

TI (“oxford house” OR “oxford home” OR “sober living” OR “sober living ho*” OR “sober living environment” OR “recovery residence” OR “halfway house” OR “halfway residence” OR “transitional house” OR domiciliary OR “wet house” OR “dry house” ) AND TI ( recovery OR remission OR abstinence OR "harm reduction" OR “substance abuse” OR “substance misuse” OR “substance dependence” OR “drug dependence” OR “substance use disorder” OR “alcohol use disorder” OR “drug use disorder” OR alcohol* OR marijuana OR “THC” OR cannabis OR cocaine OR heroin OR opioid* OR opiate* OR narcotic* OR amphetamine* OR methamphetamine* OR benzodiazepine* OR barbiturate* OR hallucinogen* OR inhalant* OR steroid* OR “club drug*” OR ecstasy OR “MDMA” OR stimulant* OR cost-benefit OR cost-offset OR cost-effectiveness OR “cost benefit” OR “cost offset” OR “cost effectiveness”)

**CENTRAL (Cochrane Registry)**

Same as for CINAHL

**PsycINFO**

Same as for CINAHL

**F. Clinical models of continuing care**

**Pubmed**

((“continuing care”[Title/Abstract] OR aftercare[Title/Abstract] OR “recovery monitoring”[Title/Abstract] OR “recovery management check up”[Title/Abstract] OR “recovery management check-up”[Title/Abstract] OR “recovery management checkup”[Title/Abstract] OR re-intervention[Title/Abstract]) AND ((recovery[Title/Abstract] OR remission[Title/Abstract] OR abstinence[Title/Abstract] OR "harm reduction"[Title/Abstract] OR “substance abuse”[Title/Abstract] OR “substance misuse”[Title/Abstract] OR “substance dependence”[Title/Abstract] OR “drug dependence”[Title/Abstract] OR “substance use disorder”[Title/Abstract] OR “alcohol use disorder”[Title/Abstract] OR “drug use disorder”[Title/Abstract] OR alcohol*[Title/Abstract] OR marijuana[Title/Abstract] OR “THC”[Title/Abstract] OR cannabis[Title/Abstract] OR cocaine[Title/Abstract] OR heroin[Title/Abstract] OR opioid*[Title/Abstract] OR opiate*[Title/Abstract] OR narcotic*[Title/Abstract] OR amphetamine*[Title/Abstract] OR methamphetamine*[Title/Abstract] OR benzodiazepine*[Title/Abstract] OR barbiturate*[Title/Abstract] OR hallucinogen*[Title/Abstract] OR inhalant*[Title/Abstract] OR steroid*[Title/Abstract] OR “club drug*”[Title/Abstract] OR ecstasy[Title/Abstract] OR “MDMA”[Title/Abstract] OR stimulant*[Title/Abstract] OR cost-benefit[Title/Abstract] OR cost-offset[Title/Abstract] OR cost-effectiveness[Title/Abstract]) OR “cost benefit”[Title/Abstract] OR “cost offset”[Title/Abstract] OR “cost effectiveness”[Title/Abstract]))

**Embase**

(‘continuing care’:ab,ti OR aftercare:ab,ti OR ‘recovery monitoring’:ab,ti OR ‘recovery management check up’:ab,ti OR ‘recovery management check-up’:ab,ti OR ‘recovery management checkup’:ab,ti OR re-intervention:ab,ti) AND (recovery:ab,ti OR remission:ab,ti OR abstinence:ab,ti OR 'harm reduction':ab,ti OR ‘substance abuse’:ab,ti OR ‘substance misuse’:ab,ti OR ‘substance dependence’:ab,ti OR ‘drug dependence’:ab,ti OR ‘substance use disorder’:ab,ti OR ‘alcohol use disorder’:ab,ti OR ‘drug use disorder’:ab,ti OR alcohol*:ab,ti OR marijuana:ab,ti OR ‘THC’:ab,ti OR cannabis:ab,ti OR cocaine:ab,ti OR heroin:ab,ti OR opioid*:ab,ti OR opiate*:ab,ti OR narcotic*:ab,ti OR amphetamine*:ab,ti OR methamphetamine*:ab,ti OR benzodiazepine*:ab,ti OR barbiturate*:ab,ti OR hallucinogen*:ab,ti OR inhalant*:ab,ti OR steroid*:ab,ti OR ‘club drug*’:ab,ti OR ecstasy:ab,ti OR ‘MDMA’:ab,ti OR stimulant*:ab,ti OR cost-benefit:ab,ti OR cost-offset:ab,ti OR cost-effectiveness:ab,ti OR ‘cost benefit’:ab,ti OR ‘cost offset’:ab,ti OR ‘cost effectiveness’:ab,ti)

**CINAHL**

AB (“continuing care” OR aftercare OR “recovery monitoring” OR “recovery management check up” OR “recovery management check-up” OR “recovery management checkup” OR re-intervention) AND AB (recovery OR remission OR abstinence OR "harm reduction" OR “substance abuse” OR “substance misuse” OR “substance dependence” OR “drug dependence” OR “substance use disorder” OR “alcohol use disorder” OR “drug use disorder” OR alcohol* OR marijuana OR “THC” OR cannabis OR cocaine OR heroin OR opioid* OR opiate* OR narcotic* OR amphetamine* OR methamphetamine* OR benzodiazepine* OR barbiturate* OR hallucinogen* OR inhalant* OR steroid* OR “club drug*” OR ecstasy OR “MDMA” OR stimulant* OR cost-benefit OR cost-offset OR cost-effectiveness OR “cost benefit” OR “cost offset” OR “cost effectiveness”)

AB (“continuing care” OR aftercare OR “recovery monitoring” OR “recovery management check up” OR “recovery management check-up” OR “recovery management checkup” OR re-intervention) AND TI (recovery OR remission OR abstinence OR "harm reduction" OR “substance abuse” OR “substance misuse” OR “substance dependence” OR “drug dependence” OR “substance use disorder” OR “alcohol use disorder” OR “drug use disorder” OR alcohol* OR marijuana OR “THC” OR cannabis OR cocaine OR heroin OR opioid* OR opiate* OR narcotic* OR amphetamine* OR methamphetamine* OR benzodiazepine* OR barbiturate* OR hallucinogen* OR inhalant* OR steroid* OR “club drug*” OR ecstasy OR “MDMA” OR stimulant* OR cost-benefit OR cost-offset OR cost-effectiveness OR “cost benefit” OR “cost offset” OR “cost effectiveness”)

TI (“continuing care” OR aftercare OR “recovery monitoring” OR “recovery management check up” OR “recovery management check-up” OR “recovery management checkup” OR re-intervention) AND AB (recovery OR remission OR abstinence OR "harm reduction" OR “substance abuse” OR “substance misuse” OR “substance dependence” OR “drug dependence” OR “substance use disorder” OR “alcohol use disorder” OR “drug use disorder” OR alcohol* OR marijuana OR “THC” OR cannabis OR cocaine OR heroin OR opioid* OR opiate* OR narcotic* OR amphetamine* OR methamphetamine* OR benzodiazepine* OR barbiturate* OR hallucinogen* OR inhalant* OR steroid* OR “club drug*” OR ecstasy OR “MDMA” OR stimulant* OR cost-benefit OR cost-offset OR cost-effectiveness OR “cost benefit” OR “cost offset” OR “cost effectiveness”)

TI (“continuing care” OR aftercare OR “recovery monitoring” OR “recovery management check up” OR “recovery management check-up” OR “recovery management checkup” OR re-intervention) AND TI (recovery OR remission OR abstinence OR "harm reduction" OR “substance abuse” OR “substance misuse” OR “substance dependence” OR “drug dependence” OR “substance use disorder” OR “alcohol use disorder” OR “drug use disorder” OR alcohol* OR marijuana OR “THC” OR cannabis OR cocaine OR heroin OR opioid* OR opiate* OR narcotic* OR amphetamine* OR methamphetamine* OR benzodiazepine* OR barbiturate* OR hallucinogen* OR inhalant* OR steroid* OR “club drug*” OR ecstasy OR “MDMA” OR stimulant* OR cost-benefit OR cost-offset OR cost-effectiveness OR “cost benefit” OR “cost offset” OR “cost effectiveness”)

**CENTRAL (Cochrane Registry)**

Same as for CINAHL

**PsycINFO**

Same as for CINAHL

**Update Rapid Scoping Review March 2022**

Using the terms defined by the Kelly review, we worked with library specialists to develop search strategies to cover 5 of the forms of RSS (1. Clinical Models of Continuing Care; 2. Peer Based Recovery Support Services; 3. Recovery housing; 4. Recovery Community Centres; 5. Recovery Support Services in Educational Settings). In addition, employment services targeted at people with AOD use disorders were added as a sixth category.

**MEDLINE**

| 1. exp Aftercare/ | 237829 |
| --- | --- |
| 2. exp *Substance-Related Disorders/rh | 15255 |
| 3. Substance Abuse Treatment Centers/ | 5454 |
| 4. recovery.tw. | 584103 |
| 5. remission.tw. | 148308 |
| 6. (abstinence or abstain*).tw,kf. | 33516 |
| 7. (harm* adj3 reduc*).tw,kf. | 15302 |
| 8. (substance adj3 (abus* or misus* or "use" or dependen*)).tw,kf. | 87983 |
| 9. (drug adj3 (abus* or misus* or "use" or dependen*)).tw,kf. | 111778 |
| 10. (("substance use" or "drug use") adj3 disorder*).tw,kf. | 25586 |
| 11. alcohol*.tw,kf. | 425117 |
| 12. cost-benefit.tw,kf. | 14123 |
| 13. cost-offset.tw,kf. | 204 |
| 14. cost-effectiveness.tw,kf. | 82750 |
| 15. (Recover* adj3 (support* or coach*)).tw. | 5427 |
| 16. (peer* adj3 (support* or coach* or recover* or mentor* or based or model or led)).tw. | 17732 |
| 17. exp *Peer Group/ | 11012 |
| 18. *Mentors/ | 7066 |
| 19. (Recover* adj3 cent*).tw. | 2486 |
| 20. (Recover* adj3 communit*).tw. | 1630 |
| 21. (Peer adj3 participat*).tw. | 778 |
| 22. *Community Health Services/ | 20798 |
| 23. *Group Homes/ | 627 |
| 24. *Halfway Houses/ | 606 |
| 25. *Community Support/ | 38 |
| 26. ((colleg* or school* or universit*) adj3 recovery).tw,kf. | 308 |
| 27. *Universities/ | 17634 |
| 28. *Students/ | 42492 |
| 29. (sober adj3 (living or hous* or home*)).tw. | 108 |
| 30. ((halfway or half way) adj3 (hous* or Home* or residen*)).tw. | 319 |
| 31. (oxford adj3 (hous* or home*)).tw. | 152 |
| 32. (transitional adj3 (hous* or home*)).tw. | 338 |
| 33. wet house.tw. | 2 |
| 34. dry house.tw. | 1 |
| 35. (recovery adj3 (hous* or home* or residen*)).tw. | 1586 |
| 36. continuing care.tw. | 1922 |
| 37. (recovery adj3 (monitor* or manag* or check-up or checkup)).tw. | 4180 |
| 38. re-intervention*.tw. | 3603 |
| 39. aftercare.tw. | 3884 |
| 40. exp Employment/ | 103522 |
| 41. *Rehabilitation,Vocational/ | 5374 |
| 42. (support* adj3 (employment or work or placement)).tw. | 18822 |
| 43. 1 or 2 or 3 or 4 or 5 or 6 or 7 or 8 or 9 or 10 or 11 | 1533914 |
| 44. 12 or 13 or 14 | 94500 |
| 45. 15 or 16 or 17 or 18 | 37789 |
| 46. 19 or 20 or 21 or 22 | 25627 |
| 47. 23 or 24 or 29 or 30 or 31 or 32 or 33 or 34 or 35 | 3359 |
| 48. 25 or 26 or 27 or 28 | 56006 |
| 49. 36 or 37 or 38 or 39 | 13543 |
| 50. 40 or 41 or 42 | 123520 |
| 51. 45 or 46 or 47 or 48 or 49 or 50 | 254324 |
| 52. 43 and 51 | 42408 |
| 53. 52 and 44 | 390 |
| 54. limit 53 to yr="2017 - 2022" | 158 |

**EMBASE**

| 1. ('Continuing Care' or 'Aftercare' or 'Recovering Monitor*' or 'Recovery Management Check*' or 're-intervention').ti,ab,kw. | 14512 |
| --- | --- |
| 2. ('recovery coach*' or 'peer recovery support' or 'peer-based recovery support service' or 'individual peer support' or 'peer mentor*' or 'peer support worker').ti,ab,kw. | 2100 |
| 3. ('recovery community center' or 'recovery center' or 'recovery support center' or 'peer support center' or 'recovery community organization' or 'peer participatory model').ti,ab,kw. | 225 |
| 4. ('collegiate recovery' or 'collegiate recovery program' or 'recovery school' or 'recovery high school' or 'university recovery centre').ti,ab,kw. | 68 |
| 5. ('oxford ho*' or 'Sober living' or 'sober living ho*' or 'sober living environment' or 'recovery residence' or 'halfway house' or 'transitional house' or 'domiciliary' or 'dry house').ti,ab,kw. | 4352 |
| 6. ('employment' or 'vocational rehabilitation' or 'supported employment' or 'placement').ti,ab,kw. | 348977 |
| 7. *alcohol abuse/ | 6946 |
| 8. exp substance abuse/dt, rh, th [Rehabilitation, Therapy] | 330 |
| 9. exp drug abuse/pc, rh, th [Prevention, Rehabilitation, Therapy] | 984 |
| 10. exp drug dependence/rh, th [Rehabilitation, Therapy] | 22647 |
| 11. ((substance or drug* or alcohol) adj3 (misuse or abuse or addiction)).ab,ti. | 126944 |
| 12. ((substance or drug) adj3 user*).ab,ti. | 25850 |
| 13. ('substance abuse disorder' or 'alcohol abuse disorder' or 'alcoholic').ti,ab. | 109143 |
| 14. (preventative or rehabilit* or therapy or support or service).ti,ab,kw. | 5598206 |
| 15. recovery.ab. or recovery.ti. or recovery.kw. | 773001 |
| 16. (peer or social or community).ti,ab,kw. | 1750327 |
| 17. 1 or 2 or 3 or 4 or 5 or 6 | 368942 |
| 18. 7 or 11 or 12 or 13 | 255646 |
| 19. (preventative or rehabilit* or therapy or support or service).ti,ab,kw. | 5598206 |
| 20. 18 and 19 | 46829 |
| 21. 8 or 9 or 10 | 23649 |
| 22. 20 or 21 | 68380 |
| 23. 17 and 22 | 2430 |
| 24. limit 23 to (human and english language and yr="2017 - 2022") | 650 |

**PsycINFO**

| 1. ('Continuing Care' or 'Aftercare' or 'Recovering Monitor*' or 'Recovery Management Check*' or 're-intervention').ti. or ('Continuing Care' or 'Aftercare' or 'Recovering Monitor*' or 'Recovery Management Check*' or 're-intervention').ab. or ('Continuing Care' or 'Aftercare' or 'Recovering Monitor*' or 'Recovery Management Check*' or 're-intervention').id. | 3771 |
| --- | --- |
| 2. ('recovery coach*' or 'peer recovery support' or 'peer-based recovery support service' or 'individual peer support' or 'peer mentor*' or 'peer support worker').ti. or ('recovery coach*' or 'peer recovery support' or 'peer-based recovery support service' or 'individual peer support' or 'peer mentor*' or 'peer support worker').ab. or ('recovery coach*' or 'peer recovery support' or 'peer-based recovery support service' or 'individual peer support' or 'peer mentor*' or 'peer support worker').id. | 1428 |
| 3. ('recovery community center' or 'recovery center' or 'recovery support center' or 'peer support center' or 'recovery community organization' or 'peer participatory model').ti. or ('recovery community center' or 'recovery center' or 'recovery support center' or 'peer support center' or 'recovery community organization' or 'peer participatory model').ab. or ('recovery community center' or 'recovery center' or 'recovery support center' or 'peer support center' or 'recovery community organization' or 'peer participatory model').id. | 92 |
| 4. ('collegiate recovery' or 'collegiate recovery program' or 'recovery school' or 'recovery high school' or 'university recovery centre').ti. or ('collegiate recovery' or 'collegiate recovery program' or 'recovery school' or 'recovery high school' or 'university recovery centre').ab. or ('collegiate recovery' or 'collegiate recovery program' or 'recovery school' or 'recovery high school' or 'university recovery centre').id. | 135 |
| 5. ('oxford ho*' or 'Sober living' or 'sober living ho*' or 'sober living environment' or 'recovery residence' or 'halfway house' or 'transitional house' or 'domiciliary' or 'dry house').ti. or ('oxford ho*' or 'Sober living' or 'sober living ho*' or 'sober living environment' or 'recovery residence' or 'halfway house' or 'transitional house' or 'domiciliary' or 'dry house').ab. or ('oxford ho*' or 'Sober living' or 'sober living ho*' or 'sober living environment' or 'recovery residence' or 'halfway house' or 'transitional house' or 'domiciliary' or 'dry house').id. | 962 |
| 6. ('employment' or 'vocational rehabilitation' or 'supported employment').ti. or ('employment' or 'vocational rehabilitation' or 'supported employment').ab. or ('employment' or 'vocational rehabilitation' or 'supported employment').id. | 63997 |
| 7. (drug* or alcohol* or substance*).ti,ab,id. | 419899 |
| 8. (remission or sustain* or effective* or efficacy or measure* or compari* or rate* or retention or sustain* or improve* or reduc* or benchmark* or analysis or engage* or 'quality of life' or happiness or impact or assess*).ti,ab,id. | 3352302 |
| 9. (1 or 2 or 3 or 4 or 5 or 6) and 7 and 8 | 6044 |
| 10. (cost* or value or ROI or spend* or economic* or return).ti,ab,id. | 492176 |
| 11. 9 and 10 | 1015 |
| 12. limit 11 to (human and english language and yr="2017 -2022") | 260 |
